# Skin simulants for wound ballistic investigation – an experimental study

**DOI:** 10.1007/s00414-024-03223-1

**Published:** 2024-04-03

**Authors:** Victoria K. S. Fischer, Markus A. Rothschild, Beat P. Kneubuehl, Thomas Kamphausen

**Affiliations:** 1 Institute of Legal Medicine, Faculty of Medicine, University of Cologne, Melatenguertel 60/62, 50823 Cologne, Germany; 2 Bpk Consultancy GmbH, Forstweg 25, 3603 Thun, Switzerland

**Keywords:** Wound ballistics, Gunshot, Skin simulant, Reconstruction, Dental silicone, Gelatine

## Abstract

Gunshot wound analysis is an important part of medicolegal practice, in both autopsies and examinations of living persons. Well-established and studied simulants exist that exhibit both physical and biomechanical properties of soft-tissues and bones. Current research literature on ballistic wounds focuses on the biomechanical properties of skin simulants. In our extensive experimental study, we tested numerous synthetic and natural materials, regarding their macromorphological bullet impact characteristics, and compared these data with those from real bullet injuries gathered from medicolegal practice. Over thirty varieties of potential skin simulants were shot perpendicularly, and at 45°, at a distance of 10 m and 0.3 m, using full metal jacket (FMJ) projectiles (9 × 19 mm Luger). Simulants included ballistic gelatine at various concentrations, dental silicones with several degrees of hardness, alginates, latex, chamois leather, suture trainers for medical training purposes and various material compound models. In addition to complying to the general requirements for a synthetic simulant, results obtained from dental silicones shore hardness 70 (backed with 20 % by mass gelatine), were especially highly comparable to gunshot entry wounds in skin from real cases. Based on these results, particularly focusing on the macroscopically detectable criteria, we can strongly recommend dental silicone shore hardness 70 as a skin simulant for wound ballistics examinations.

**Supplementary information:**

The online version contains supplementary material available at 10.1007/s00414-024-03223-1.

## Introduction

Terminal ballistic research includes the field of wound ballistics, a specialized biomechanical branch evaluating wound effects in biological tissues (especially of human individuals). The morphology of gunshot wounds allows to infer specific properties of the wound-causing projectiles, which can be of great forensic importance. Characteristics of the entry wound, the direction and angle of the shot, the trajectory of the projectile through the tissue (wound channel) and the distance between the firearm and its target are, therefore, essential forensic investigation factors. Medical examination, technical analyses, and experimental reconstructions in specific cases, looking back on single events or derived as general statements, can provide new insights.

Reliable simulants representing soft tissues, including skin, are required for wound ballistic investigations and research. The use of materials from human cadavers [1–8], living animals [9–11], or animal parts [12–14] has been described in the literature. Due to ethical considerations, the use of simulants made of synthetic or natural materials are preferable. Ballistic gelatine or ballistic glycerin soap are commonly used as such soft body tissue simulants [11, 15–22]. Despite a considerable amount of experience gained in the preparation and use of both substances as soft tissues simulants over decades, certain specific questions still require the development of modified simulants for special tissues (e. g. brain, lung) [23, 24]. Bone models, comprising polyurethane (e. g. Synbone®), have given rise to composite models wound ballistic research [19, 25, 26].

Skin simulant studies have been published that focus on aiding forensic examinations, such as sharp and blunt force trauma [27–35]. However, the morphology of skin-penetrating gunshot wounds currently use human and animal cadaver skin for ballistic wound investigations [3, 36–39]. Although several relevant studies on natural and synthetic skin simulants exist, their focus is predominantly on the biomechanical properties of the simulants, such as tensile strength and shore hardness [40–45].In 2005, Jussila argued that the properties of a tissue (skin) simulant do not have to exactly match those of comparable living tissue [40]. To the best of our knowledge, our study is the first to report exclusively on the macroscopic comparability of bullet entry wounds created in skin simulants to those in human skin. This study focuses on identifying a skin simulant which exhibits bullet entry defects comparable to human skin in terms of visually determinable parameters—of importance when reconstructing the ballistic path and defining a skin defect as an entry wound.

**Fig. 1:**
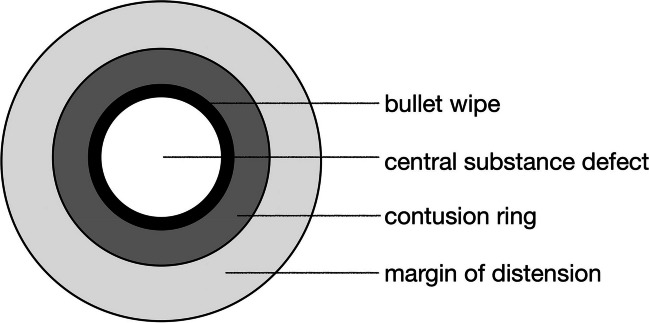
Morphologic terminology of a gunshot entry wound (perpendicular distant shot with a full metal jacket bullet)

Full metal jacket projectiles cause specific, recurring, patterns and effects indicative of an entry wound (Fig. 1). Sellier [46] and Kneubuehl [20] showed that skin, when impacted by a projectile, is only minimally formed into a funnel shape due to inertia. After exceeding the limit of its expansibility, the skin ruptures, the projectile penetrates and, due to the projectile’s high kinetic energy, the skin is “crushed”, producing a non-adaptable, hole-shaped defect. The defect is surrounded by a circular epithelial abrasion and small radial tears often persist, as the surrounding tissue is accelerated radially and away from the bullet, commencing the expansion of the temporal cavity. Due to the elasticity of the skin, a bullet wound resulting from perpendicular entry is round and often slightly narrower than the bullet´s caliber. By comparison, the effect of a bullet with a sloped trajectory results in oval defects of slightly larger diameter than the bullet´s caliber. When a projectile impacts uncovered skin, gunshot residues, oil and metallic abrasions adhering to the projectile can be sloughed off onto the skin, leaving a bullet wipe surrounding the central skin defect.

## Materials

### Gelatine

#### Gelatine blocks

Gelatine blocks 40 x 20 x 20 cm were prepared with gelatine grade 3 (GELITA Deutschland GmbH, Germany) from the same calibrated batch, to the gelatine preparation method provided by Jussila (2004) [16] with the difference that a gelatine-water-mixture 20 % gelatine (by mass) instead of 10 % was used. This was stored at 4 °C until used within 24-48 h. Gelatine blocks were used as soft tissue simulant to which the skin simulants tested herein were attached.

#### Gelatine sheets

Gelatine sheets of 0.3 cm and 0.6 cm thickness were produced as skin simulants with coarse-grained gelatine grade 1 (GELITA Deutschland GmbH, Germany) from the same calibrated batch, based on the recommendations of Jussila (2004) but in increasing concentrations from 10 % to 60 % gelatine (by mass) in increments of ten [16]. The sheets were fixed to the gelatine blocks by means of a 20 % gelatine (by mass) liquid layer (serving as glue) and then shot perpendicularly and from an angle of 45°, from distances of 10 m and 0.3 m.

### Dental silicone

Dental silicones of increasing degrees of hardness (shore hardness 16, 22, 32, 60, 70 and 85) were tested. Vinylpolysiloxane (addition curing silicone) duplicating material “Elite double fast” in shore hardness 16, 22 and 32 (Zhermack S.p.A., Italy) was prepared according to the manufacturer's instructions – each of the two fluid components in a mixture ratio of base : catalyst = 1 : 1 (mixing time 30 sec.) and then poured and formed into sheets of 0.05 cm and 0.6 cm thickness (processing time 5 – 10 min). Kneadable addition curing silicones in shore hardness 60 and 70 (Briegeldental, Germany) and 85 (Zhermack S.p.A., Italy) were prepared according to the manufacturer´s instructions – each of the two components in a mixture ratio of base : catalyst = 1 : 1 were kneaded for 30 sec. to a homogenous mass and then rolled out into flat sheets of 0.15 cm and 0.3 cm thickness (processing time 1:20 – 2:00 min). All dental silicone sheets thus prepared were attached to the gelatine block with molten gelatine 20 % (by mass) and then perpendicular shots were fired from 10 m and 0.3 m. The silicone shore hardness 70 was additionally shot from an angle of 45°.

#### Dental silicone-chamois leather-compound

A thin layer (thickness 0.15 cm) of kneadable silicone shore hardness 70 (Briegeldental, Germany) was fixed on a sheet of chamois leather skin (thickness 0.1 cm) by using a very thin layer (layer thickness < 0.05 cm) of fluid addition-curable silicone shore hardness 22 (Zhermack S.p.A., Italy). The chamois leather was placed directly on a solidifying gelatine block and shots were subsequently fired perpendicularly and obliquely at an angle of 45° from 10 m and 0.3 m.

In addition, silicone shore hardness 22 was applied directly to the chamois leather in the thinnest possible layer with a spatula (layer thickness < 0.05 cm). The excess silicone was removed from the leather. It was placed directly on the solidifying gelatine block (chamois leather on gelatine). Perpendicular and oblique shots (45°) were delivered from distances of 10 m and 0.3 m.

#### Dental silicone-artificial leather-compound

A thin layer (thickness 0.15 cm) of kneadable silicone shore hardness 70 (Briegeldental, Germany) was fixed on a sheet of artificial leather skin (thickness 0.1 cm) using an ultra-thin layer of fluid silicone shore hardness 22 (Zhermack S.p.A., Italy). The compound was placed directly on the solidifying gelatine block (artificial leather on gelatine), which was subsequently fired at perpendicularly and obliquely at an angle of 45° from 10 m and 0.3 m.

### Chamois leather

Chamois leather in a thickness of about 0.1 cm was placed directly on the surface of the solidifying gelatine block. Perpendicular shots were delivered from 10 m and 0.3 m.

#### Chamois leather with Ballistol® multi-purpose oil

Chamois leather, thickness of 0.1 cm, was placed directly on the surface of the solidifying gelatine block and then evenly wetted with “Ballistol® Universalöl Spray” (BALLISTOL, Germany). Perpendicular shots were delivered from 10 m and 0.3 m.

#### Chamois leather with beeswax

100 % pure beeswax was heated in a water bath until completely melted. The liquid wax was applied in the thinnest possible layer (layer thickness < 0.05 cm) on the chamois leather, the excess wax was removed with a heated spatula. Furthermore, chamois leather was placed in liquefied pure beeswax until it was completely soaked. The excess beeswax was removed from both sides of the leather using a heated spatula until no wax remained adhering to the spatula. The single-side-waxed chamois leather was then directly laminated onto the gelatine block with a small amount of melted 20 % gelatine (by mass). The double-side-waxed chamois leather was attached to a gelatine block with adhesive tape. Perpendicular shots were delivered from 10 m and 0.3 m distances.

### Dental alginate

Chromatic alginate impression material „Tropicalgin” and high precision alginate impression material “Neocolloid” (Zhermack S.p.A., Italy) were prepared according to the manufacturer´s instructions – each alginate impression material was mixed with water in a mixture ratio of alginate : water = 1 : 2. These were stirred for 45 sec. until a homogenous mass was formed and then rolled out into thin even sheets of 0.05 cm and 0.3 cm thickness (“Tropicalgin processing time” 1:35 min, setting time 2:35 min; “Neocolloid” processing time 2:00 min, setting time 3:30 min). The alginate sheets were stored in an airtight humidity chamber, to prevent moisture loss, until they were attached directly to the gelatine block with adhesive tape and shot perpendicularly from 10 m and 0.3 m.

### Skin simulants for medical suture training proposes

In this study the “Transparent Intracutaneous Suture Pad for use with trainer 7060” (Erler-Zimmer, Germany), the “RealSuture 1-layer translucent suture pad” (Erler-Zimmer, Germany), the “Double-sided Skin Suture Pad” (3B Scientific, Germany) and the “Life/Form Replacement Suture Pad – Light Skin” (Nasco, USA) were attached directly to the gelatine block with adhesive tape. Perpendicular shots were delivered from 10 m and 0.3 m.

### Latex

Natural low ammonia latex milk with over 60 % solid content (Latex-24, Germany) was poured onto a flat ceramic surface in the thinnest possible layer (layer thickness < 0.05 cm). After drying at ambient temperature, this step was repeated twice to obtain a latex skin of about 0.1 cm. The latex was then fixed to the gelatine block by means of 20 % gelatine (by mass). Perpendicular shots from 10 m and 0.3 m were delivered.

## Methods

### Firearm and ammunition

The shots were fired using a SIG Sauer P226 X-Five Allround pistol, cal. 9 x 19 mm Luger (SIG Sauer, Eckernfoerde, Germany) and 9 x 19 mm Luger full metal jacket (FMJ) round head ammunition, weight: 8.0 g/124 grs (Sellier & Bellot, Czech Republic). Basic ballistic parameters v_1m_, E_1m_, v_10m_ and E_10m_ of the weapon-ammunition combination were collected using an infrared photoelectric sensor DRELLO IMS 8500-1 (DRELLO, Germany) by shooting 10 rounds prior to the main experiments on simulants tested in this study.

### Analysis

The skin simulants listed above were each prepared as described and fixed to the gelatine blocks. Subsequently, perpendicular shots were fired at the models from 10 m and 0.3 m (n=10 each) to imitate a long-range shot and a relatively close-range shot. The morphology of the bullet entry defects was photographed and macroscopically independently examined by three expert forensic pathologists using characteristic criteria of bullet entry wounds (central substance defect, circular contusion ring and radial tears, bullet wipe) after perpendicular (and oblique) impact of full metal jacket projectiles on uncovered skin. In addition, the gunshot entry defect in the skin simulants were compared with those in human skin from comparable real cases. Skin simulants exhibiting characteristic bullet impact features and bullet impact defects, comparable to real bullet injuries, were subsequently shot at from an angle of 45° from distances of 10 m and 0.3 m (n=10 each) and the results were evaluated macroscopically, as described above.

## Results

### Basic ballistic parameters

The velocity of the projectiles measured at distances of 1 m (v_1m_) and 10 m (v_10m_) from the muzzle was 367 m/s and 347 m/s on average, respectively.

According to Eq. (1)

1 $${E}_{k}=\frac{1}{2}m{v}^{2}$$

E_k_: kinetic energy, m: mass, v: velocity

the average kinetic energies E_1m_ and E_10m_ of the projectiles were calculated to be 542 J and 481 J, respectively.

### Ballistic gelatine

**Fig. 2:**
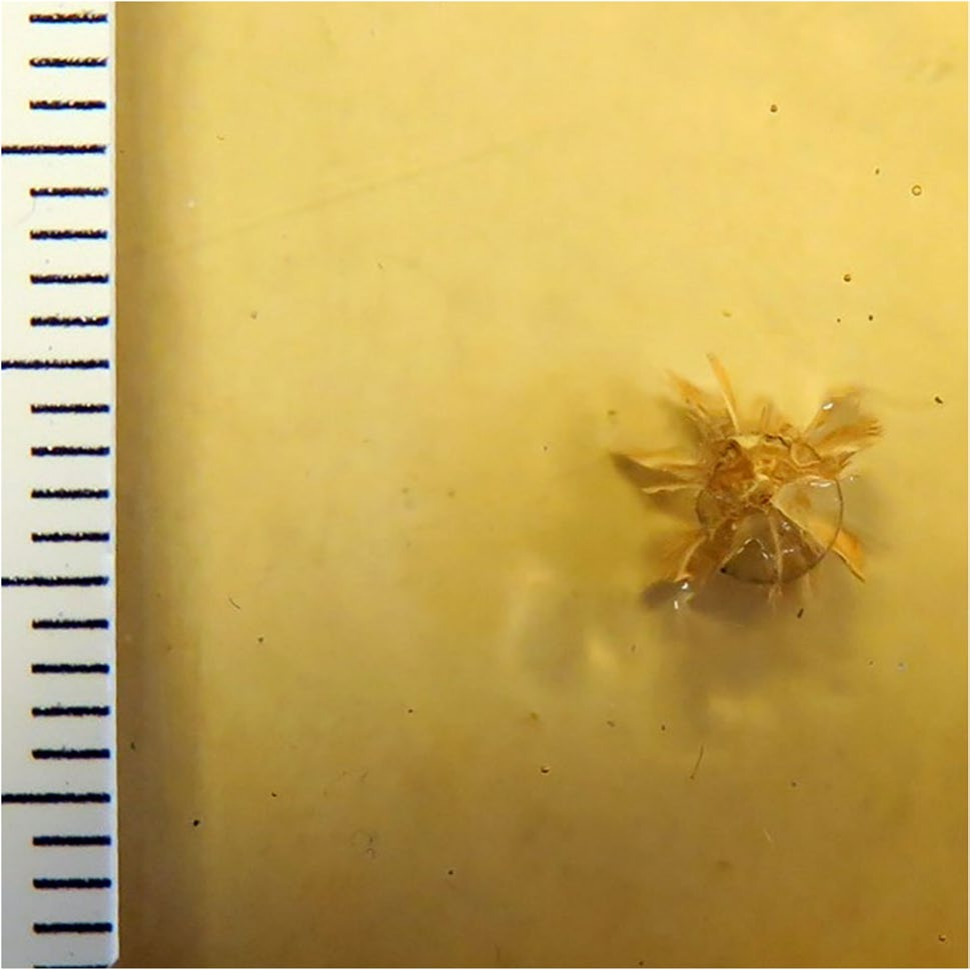
Gelatine 40 % (by mass) (thickness 0.3 cm, perpendicular shot, 10 m distance)

Irrespective of gelatine concentration, layer thickness and shooting range, a central substance defect with a maximum diameter of 0.2 cm (perpendicular shots), or 0.4 × 0.6 cm (angled shots), was detected in all gelatine sheets. A further superficial material defect, comparable to a contusion ring, was observed in all gelatine concentrations and layer thicknesses around the central substance defect, after perpendicular shots. The contusion ring was crescent-shaped, pointing in the direction of the shooter, after oblique shots. The diameter of the contusion ring increased with the concentration of the gelatine, with diameters of 0.3 cm to 0.5 cm observed after perpendicular shots. Radial tears of max. 0.5 cm length were detectable around the central substance defect, their length being inversely correlated with increasing concentration of the gelatine. For gelatine concentrations at, or above, 40 % (by mass), a bullet wipe was visible and increasingly pronounced with increasing concentration levels (Fig. 2, ESM-Fig. 1–11).

### Dental silicones

Simulants containing kneadable silicones of shore hardness 60, 70 and 85 in 0.3 cm layer thickness, were characterized by a superficial central substance defect of max. 0.3 cm diameter (perpendicular shots), which tapered crater-like in the direction of the shot. Within this area, a dark bullet wipe and a superficial, circular material defect were observed. In silicones of shore hardness 60 and 85 with a layer thickness of 0.15 cm, the diameter of the central substance defects was up to 0.5 cm (perpendicular shots). A circular superficial tissue defect, comparable to a contusion ring was observable in both cases. A bullet wipe was visible on the silicone shore hardness 60, but absent on the silicone shore hardness 85. Radial tears were not detectable and shots from an angle of 45° were not carried.

Silicone shore hardness 70, with a layer thickness of 0.15 cm, showed a central substance defect with a maximum diameter of 0.4 cm after perpendicular shots, and 0.3 cm x 0.7 cm after oblique shots, irrespective of the shooting range. In each case, a bullet wipe up to 0.2 cm width was apparent — crescent-shaped in the direction of the shooter in the case of the oblique impact. Analogous to the bullet wipe, a contusion ring was visible, accompanied by radial cracks up to 0.5 cm length (after perpendicular impact) and up to 1 cm length (diagonally opposite to the shooter) after oblique impact.

The investigated silicones shore hardness 16, 22 and 32 in a layer thickness of 0.6 cm did not show a clearly detectable central substance defect (diameter < 0.1 cm) regardless of the shooting range. A dark/black bullet wipe was demarcated within a superficial material defect comparable to a contusion ring (diameter approx. 0.4 cm) located circularly around the central substance defect. Radial tears were not visible. Shots from an angle of 45° were not carried out on layers in this thickness.

**Fig. 3:**
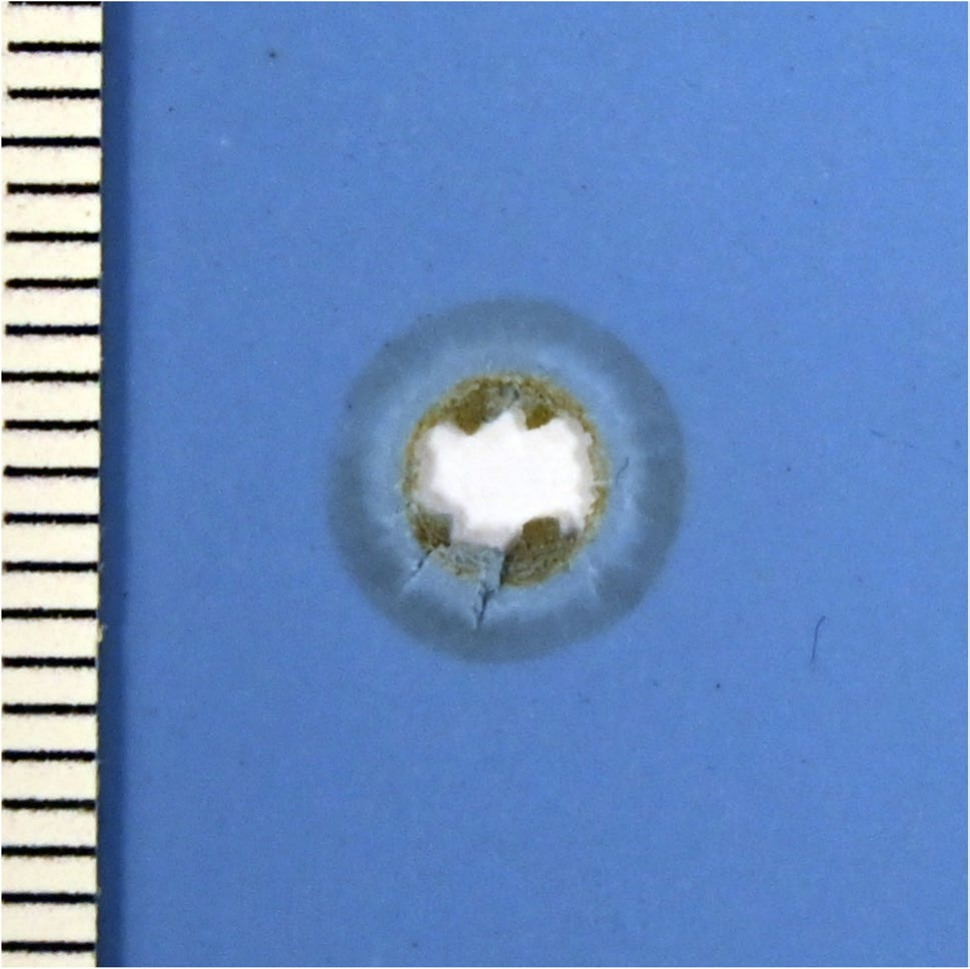
Dental silicone shore hardness 70 (thickness 0.15 cm, perpendicular shot, 10 m distance)

Simulants, each made with silicone shore hardness 16, 22 or 32 (thickness 0.05 cm) showed a central substance defect of max. 0.4 cm diameter, after perpendicular shots, and 0.5 cm x 0.3 cm, after oblique shots. The material was lifted in a bubble-like form from the gelatine beneath; the bullet wipe was mainly visible on the inner side of the central substance defect near the gelatine. At an impact angle of 45°, a crescent-shaped bullet wipe pointing in the direction of the shooter was visible. A zone of superficial material loss in a circular pattern comparable to a contusion ring around the bullet defect, was slightly pronounced (max. 0.1 cm width). Irrespective of the distance and the direction of the shot, radial tears of up to 2.5 cm length were present (Fig. 3, ESM-Fig. 12–17).

### Dental silicone (shore hardness 70)-artificial/chamois leather-compound

A sheet of 0.15 cm thick silicone shore hardness 70 was applied to the artificial leather and chamois leather by means the thinnest possible layer (layer thickness < 0.05 cm) of a silicone shore hardness 22. Following perpendicular shots, the material showed a partially punched-out central substance defect of about 0.4 cm diameter, irrespective of the shooting range. The artificial/chamois leather below showed a central substance defect of ca. 0.1 cm in diameter. The superficial material defect, comparable to a contusion ring, extended over the entire area between the central substance defect in the leather and the edge of the bullet defect in the skin simulant. Circularly adjacent to this, a narrow seam with radial tears was apparent. A central substance defect of up to approx. 0.1 cm x 0.2 cm was visible after the oblique shots. A crescent-shaped bullet wipe in the direction of the shooter of approximately 0.6 cm x 0.4 cm was visible. On the opposite side of the bullet wipe, a crescent-shaped spalling of the uppermost silicone layer up to 0.3 cm width was adjacent to the central substance defect. Radial tears were not apparent. (ESM-Fig. 18–20).

### Dental silicone (shore hardness 22)-chamois-leather-compound

**Fig. 4:**
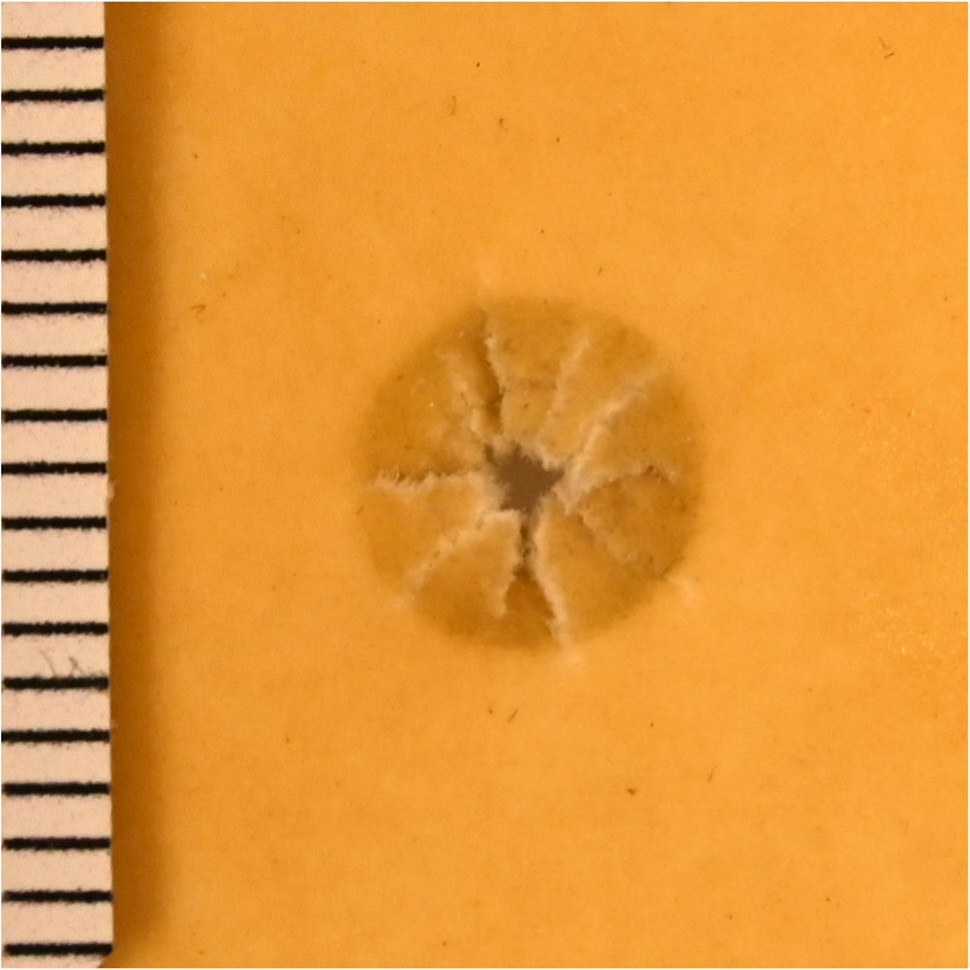
Dental silicone shore hardness 22-chamois leather-compound (perpendicular shot, 10 m distance)

The chamois leather, coated with the thinnest possible (layer thickness < 0.05 cm) layer of silicone shore hardness 22, showed central substance defects of 0.1 cm diameter at 10 m shooting range, and of 0.3 cm diameter at 0.3 m shooting range, irrespective of the angle of impact. In each case, radial tears were detected along with circular (perpendicular shots) or crescent-shaped (oblique shots) bullet wipes. Superficial material defects comparable to a contusion ring were not detectable. (Fig. 4, ESM-Fig. 21).

### Chamois leather

**Fig. 5:**
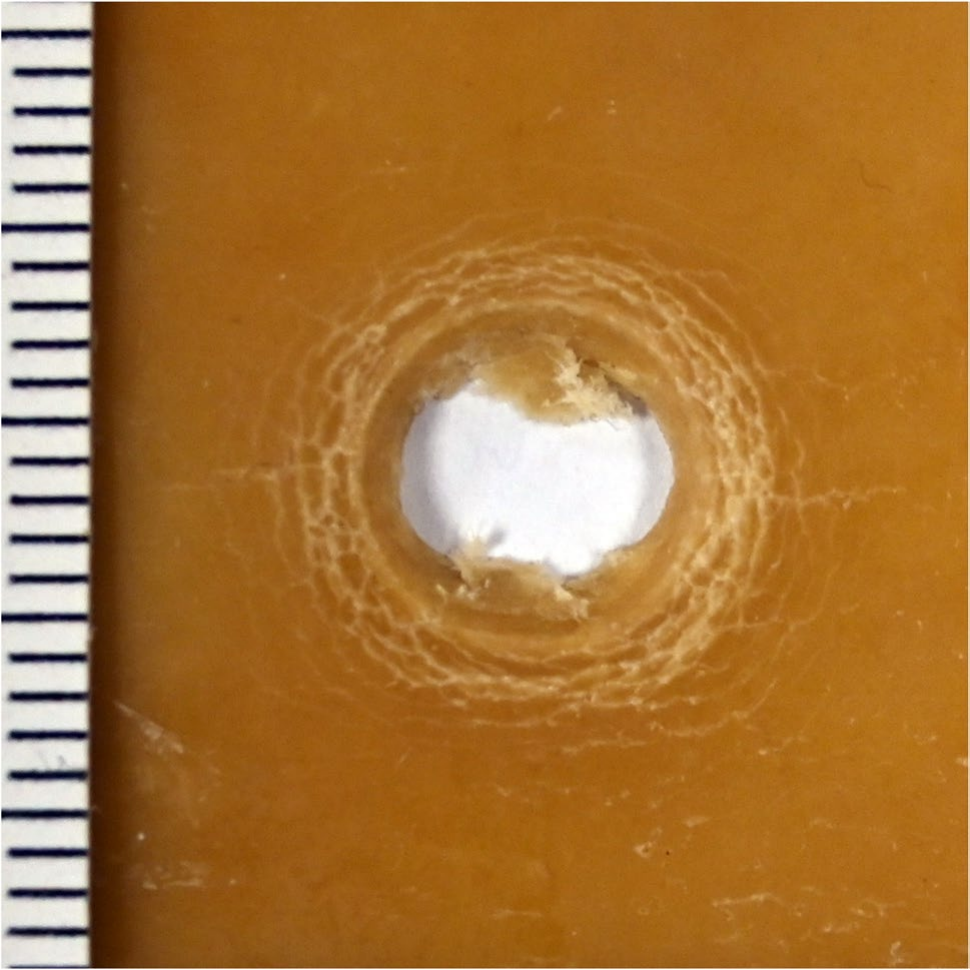
Chamois leather impregnated with beeswax (perpendicular shot, 10 m distance)

Irrespective of the shooting range, both the chamois leather impregnated with Ballistol® and the chamois leather treated with pure beeswax showed central substance defects up to the size of the calibre used, “punched out” in appearance. Circular material defects comparable to a contusion ring, radial tears, and a bullet wipe were not present. The untreated chamois leather showed central substance defects of max. 0.2 cm diameter and a bullet wipe up to 0.3 cm width. Shots from an angle of 45° were not carried out (Fig. 5, ESM-Fig. 22, 23).

### Alginates

**Fig. 6:**
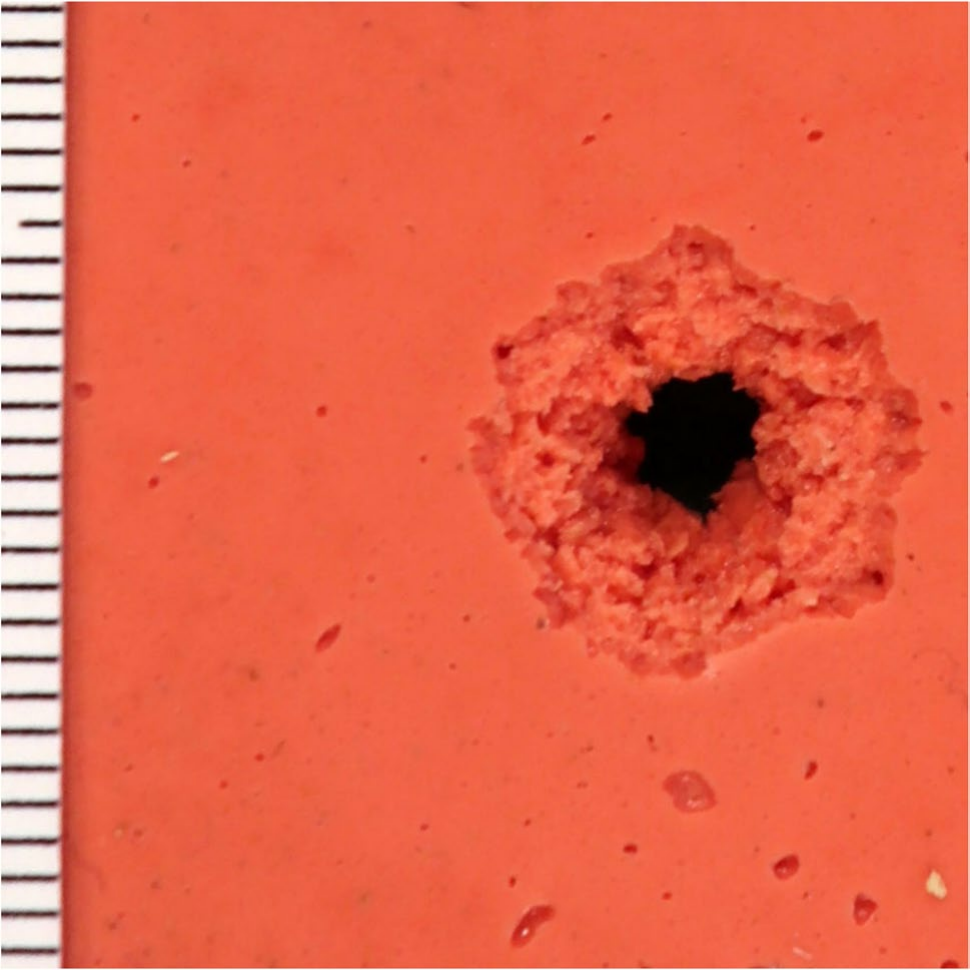
Dental alginate “Neocolloid” (thickness 0.3 cm, perpendicular shot, 10 m distance)

Independent of the shooting range, central “punched-out” tissue defects, with a maximum diameter of 0.7 cm (thickness 0.05 cm) and 0.3 cm (thickness 0.3 cm), were present. Around the central substance defect, a circular loss of substance reaching deep into the material was detectable, particularly in the thick layer alginate. A bullet wipe and radial tears were not apparent. Shots from an angle of 45° were not carried out (Fig. 6, ESM-Fig. 24).

### Skin simulants for medical training purposes

All four synthetic skin models tested for medical suture training purposes showed no visible central substance defects (diameter < 0.1 cm) visible to the naked eye, regardless of the shooting range and the angle of impact. Only the top layer of material showed a crater-like defect with a diameter of max. 0.3 cm. Radial tears and a bullet wipe were not macroscopically detectable. Shots from an angle of 45° were not performed. (ESM-Fig. 25).

### Latex

**Fig. 7:**
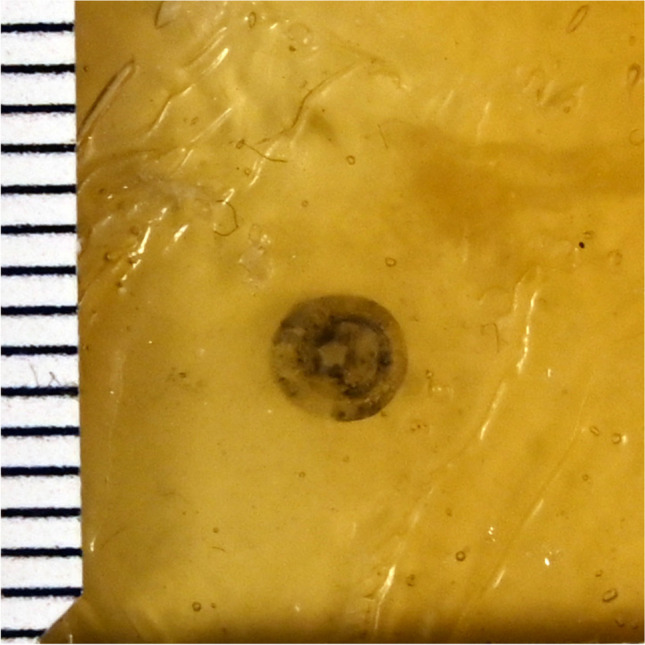
Latex (thickness 0.1 cm, perpendicular shot, 10 m distance)

The latex skin simulant showed a central substance defect with a maximum diameter of 0.1 cm, irrespective of the shooting range. No circular substance defect, comparable to a contusion ring, could be clearly delineated, but a circular bullet wipe of up to 0.2 cm wide was present. Shots from an angle of 45° were not performed. (Fig. 7).

## Discussion

Wound ballistic examinations are indispensable in forensic pathology practice, particularly in cases involving direct bullet impact to the skin. Energy transmission from the bullet to the tissue, initiated by first contact between the bullet-tip and primary target, often skin or textiles, results in tissue damage (injury). In addition to assessing the projectile’s trajectory through the body and induced injuries, the morphology of the bullet impact location is particularly important for obtaining information on, for instance, the angle of impact and the distance between the muzzle and the body.

Complex reconstructions are often necessary to address extensive forensic issues. Numerous studies exist on several simulants used to emulate various tissues, such as muscle and bone, in wound ballistics examinations. Further studies, on synthetic and natural skin simulants, address the comparability of the biomechanical properties of potential simulants to those of human or animal skin [3, 9–11, 36–39]. To the best of our knowledge, this study is the first to assess potential wound ballistic skin simulants that – from the macroscopic aspect – produce gunshot entry defects comparable to real bullet wounds encountered in casework and that fulfil the established criteria for a simulant.

Full metal jacket bullets, excluding shots from point blank range, produce typical characteristics when they impact the skin perpendicularly [20, 46–48], see (Fig. 1): The central substance defect, a hole-like tissue defect with non-adaptable wound edges. The epidermis-free zone around the central substance defect, referred to by Kneubuehl et al. (2022) [20] as a “contusion ring”, is caused by temporary increased localised pressure within the tissue, as a function of the high kinetic energy, resulting in superficial tissue particles being lifted off in a conical shape. This can lead to radial tears at the edge of the contusion ring, due to the circular overstretching, and marks the onset of the temporal cavity by radial acceleration of the tissue away from the projectile. Prior to the mechanism being understood, it was assumed that the impacting projectile superficially abraded the surrounding skin, hence the contusion ring being formerly referred to as “abrasion ring”. In the case of an obliquely impacting bullet, the contusion ring is crescent-shaped with an eccentric widening in the direction of the shooter. According to Kneubuehl et al. (2022) [20], the contusion ring may be surrounded by the margin of distension, especially on skin that covers bone. The bullet wipe (“ring of dirt”) is generated by residues on the surface of the bullet, such as gunshot particles or barrel oil, shed in a circular pattern around the bullet defect when it impacts the skin. Particles from the bullet itself may also be sloughed off. The bullet wipe can partially cover the epidermis-free zone of the contusion ring in shots fired at uncovered skin.

According to DiMaio [5] and Sellier [46], the diameter of the central substance defect of a gunshot wound does not allow for any conclusions to be drawn about the projectile’s calibre. However, it is commonly accepted that the bullet defects formed by perpendicular impact of a non-deforming full metal jacket bullet are usually smaller than the projectile’s calibre. This can be attributed to (1) the presence of elastic and collagen fibres in the dermis, which form the basis of the skin’s extensibility and retractability [49], and (2) due to the radial movement of the surrounding tissue when impacted by the entry wound bullet, meaning that only the tip of the projectile comes into contact with the skin [46]. Peonim et al. (2016) came to similar conclusions from post-mortem examinations after a public mass shooting event in Bangkok in 2010, where an M-16 and munition cal. 5.56 × 45 mm were used. In almost three quarters of the deceased victims, the entry wounds observed during autopsy were smaller than the projectiles’ calibre. Most of the detected bullet entry wounds classified as "round" had a diameter of 0.3 cm [50]. In their work, Geisenberger et al. (2022) compared the size of bullet defects in pig skin depending on their localization (front and back of the trunk). After perpendicular shots from a distance of 1.6 m with a 9 × 19 mm Luger, they measured a mean diameter of the bullet defects of 5.61 ± 0.57 mm on the anterior trunk and 3.33 ± 1.17 mm on the back of the trunk [38]. Pircher et al. (2017) compared the influence of different bullet shapes on the size of the central substance defect and the shape of the contusion ring. After perpendicular shots using calibre 0.38 special on pig skin (belly region) from 2 m, gunshot entry wound diameters of 4.43 ± 1.33 mm (round nose), 8.43 ± 1.17 mm (wadcutter), and 6.01 ± 0.84 mm (truncated cone) were observed; the contusion ring was largest with round nose bullets, and smallest with the wadcutter projectiles [51]. Thali et al. (2002) indicated the contusion ring as being "generally 1–3 mm wide" [48].

### Ballistic gelatine as skin simulant

Gelatine was chosen due to being: well researched; in common use in wound ballistic examinations; possessing physical properties and ballistic behavior that are well understood following decades of use in emulating solid soft tissues [15–18, 20]. Jussila et al. (2005), described sheets of 40 % gelatine (by mass) exhibiting a leathery consistency, thus possibly functioning as a skin simulant [40]. In their study, Hes et al. (2023) examined gelatine sheets, incorporating various gelatine concentrations between 30 % and 45%, as a possible skin simulant. They shot 4.5 mm air rifle steel balls at the sheets, concluding that gelatine at these concentrations could be “very effective and repeatable” [41].

Building on these observations, gelatine-water mixtures, in concentrations ranging from 10 % to 60 % (in increments of ten), were poured to form sheets of different thicknesses which were then fixed to a gelatine block. The production of gelatine sheets with a gelatine concentration above 40 % (by mass) was challenging, impeding the manufacturing process (e.g., due to reduced solubility), their use as a skin simulant and their use in the final product (e.g., due to interspersion with air bubbles). For these reasons, gelatine-water-mixtures with gelatine concentration levels > 60 % were omitted in pre-tests.

Regardless of the concentration or layer thickness, the formation of the gunshot entry defects in the gelatine sheets tested herein exhibited identical patterns. The maximum diameter of the central substance defect was 0.2 cm (perpendicular shots), ranging notably below values reported in the literature [38, 51] and observations from the authors’ forensic routine experience. The diameter of the visible superficial material defect, reminiscent of a contusion ring (max. 0.5 cm diameter), increased slightly with gelatine concentration. In all gelatine sheets, radial tears were detected around the central substance defect. A bullet wipe was only visible at gelatine concentrations of ≥ 40 %, for all shooting distances and angles.

Gelatine meets the criteria for a good skin simulant in terms of availability and easy handling. Individual layers of gelatine as a potential skin simulant, and blocks of 20 % gelatine (by mass) as a tissue simulant, can be easily combined to create a compound model. Difficulties in producing gelatine sheets with concentrations > 40 % (by mass), limited storage life, as well as cooled storage (refrigerator) requirement can be considered as limiting factors. Although some authors have reported gelatine at higher concentrations as having similar biomechanical properties to human skin [40, 41], the results of our investigations did not confirm full comparability with regards to macromorphological criteria of bullet entry defects. Other simulants appear more effective for exact reconstruction of a gunshot wound.

### Dental silicone as skin simulant

In 2002, Thali et al. [52] published their study on the “skin-skull-brain-model” for wound ballistic examinations. Using a silicone “cap” (not specified in detail) stretched onto a gelatine-filled sphere of a synthetic bone simulant, the authors reported results as being “comparable to the morphology of equivalent real gunshot injuries”. Based on the latter study, Falland-Cheung et al. (2015) [28] used the “skin-skull-brain-model” to investigate blunt force effects on artificial skin made of dental silicones. Furthermore, dental silicones with various degrees of hardness (shore hardness) were declared as “good alternative materials” to pig skin and human skin with regards to their mean hardness and tear strength. In addition, their observed pig skin properties did not correspond to those for human skin previously reported in the literature [28]. Pittar et al. (2018) [27] showed that duplication silicones and different polyvinylsiloxanes meet the requirements of a skin simulant in terms of durability, reproducibility and dimensional stability.

The morphology of gunshot entry wounds in the silicones of low shore hardness 16, 22 and 32 and high shore hardness 60, 70 and 85 in thick layers included in our study did not show characteristics comparable to those of real bullet wounds. However, silicones with a higher shore hardness (in particular shore hardness 70) in very thin layers (thickness 0.15 cm), backed by 20 % gelatine (by mass), exhibited bullet entry defects similar to bullet wounds as encountered in forensic casework. This differs from the results of the test series in which the silicone of shore hardness 70 was first applied to artificial leather/chamois leather and then backed by gelatine 20 % (by mass).

All silicones assessed in our study were chosen for simple, fast processing (requiring no further technical support, devices, etc.); their manufacturer’s characteristics of long-term dimensional stability, high elasticity, and tear resistance; stability at room temperature without special storage requirements; and the ready availability of standardized controlled mixtures facilitating reproducibility. Solid compounds produced with other simulants, such as polyurethane, can be combined in the construction of complex anatomical models for extensive wound ballistics studies. With regards to materials utilised in the present study, silicones backed with gelatine (20% by mass) demonstrated the best potential for use as a ballistic skin simulant.

### Dental alginates as skin simulant

Alginates had been studied as brain simulants by Falland-Cheung et al. (2016) [23]. Nonetheless, dental alginates, while readily available, are more complex to produce than dental silicones. Our results showed a central substance defect of approximately the same size as encountered in real cases. However, as previously observed [23], the alginates were brittle, with large pieces breaking away from the surface when impacted by a projectile, barring any contusion ring comparison. Thus far, firm attachment of alginates to other simulants (e.g., polyurethane, gelatine) for wound ballistic investigations has not been successful, considerably limiting their usefulness. In addition, high storage requirements (humidity chamber) and low durability, renders the material unsuitable as a skin simulant.

### Latex as skin simulant

Production of an artificial skin layer from liquid latex proved difficult, requiring an ammonium-containing latex mixture with a solid content of at least 60 % for consistent production and setting. Despite several attempts, production of a uniform, reproduceable latex layer was not successful. Results of comparative showed no resemblance to bullet entry wounds from forensic cases; hence, latex was deemed unsuitable as a ballistic skin simulant.

### Commercial medical training simulants / suture trainers as skin simulant

Manufactured for medical training purposes, these materials contained silicones whose composition and shore hardness were not precisely defined or specified by the manufacturers. The macromorphological appearance of the entry defects generated by both shots from long distance (10 m), and relatively close distance (0.3 m), did not correspond to the bullet entry wound morphology of comparable forensic cases. Central substance defects were distinctly too small, with a maximum diameter of max. 0.1 cm; bullet wipe and contusion ring were hardly detectable. Commercial suture trainers were, therefore, deemed not suitable as skin simulants.

### Chamois leather as skin simulant

Chamois leather was used in a variety of ways in our experiments. Chamois leather, thinly coated with dental silicone of shore hardness 22, exhibited a central substance defect and a visible contusion ring. Manufacture and attachment to the gelatine block was straightforward. However, chamois leather is a natural material that can be subject to major variations in material properties. Further skin simulants including chamois leather, such as: coated with pure beeswax; oiled with Ballistol®; and with dental silicone shore hardness 70, did not produce results comparable to gunshot entry wounds in forensic cases.

### Limitations

Skin wound morphology is very challenging, depending as it does on numerous factors such as the type of weapon and ammunition (e.g., in terms of caliber, shape, velocity), the shooting range and angle of impact, intermediate targets, the localization of the affected body region, general skin conditions, etc. This article only reports findings produced by a single combination of weapon system and ammunition type. Results are likely to differ when alternative ammunition types and calibers are used. In addition, gelatine 20 % (by mass) was used as backing material due to its higher (temperature) stability over the time taken for these experiments. In subsequent studies, gelatine 10 % (by mass) should be used as backing material, being most frequently used as soft tissue simulant in wound ballistic investigations. Furthermore, this study focusses on macromorphological observations of the tested skin simulants and the materials that would be principally suitable for reproducing the FMJ bullet morphology (as depicted in Fig. 1). The biomechanical properties, such as tensile strength and the mean hardness of each skin simulant, were not included in the discussion and are not the main rationale for recommending a particular skin simulant in this work. These biomechanical variables may be important for the mechanical behavior of a projectile exhibiting initial deformation and energy distribution at first contact with the target. Unfortunately, skin simulants fulfilling these requirements do not produce comparable entry wounds, which are important for shot reconstruction by medicolegal experts (e.g., forensic pathologists). Some of the tested skin simulants (e.g., alginates) failed to establish a strong bond with the gelatine block as a soft tissue simulant, which may have influenced the bullet entry defect morphology. Further investigations of possible skin simulant materials should include their biomechanical properties in combination with their macroscopic appearance, along with consideration of biological factors that can influence human skin. In addition, the influence of different weapon/ammunition systems on the macromorphology of the bullet defects should be tested.

## Conclusions

Our results show dental silicones as fulfilling most requirements for an adequate skin simulant in wound ballistic examinations. Commercial dental silicones are of consistent quality, readily available, possess favorable properties such as ease of processing, storage, durability and adaptability and are easily amalgamated with gelatine to create more complex anatomical models. Gelatine, in concentrations higher than those used in soft tissue simulant models, exhibits some visually distinguishable aspects typical of gunshot entrance wound criteria, but is an insufficient match to forensic gunshot wounds. Evaluation of data, regarding macroscopic bullet impact criteria, identified silicone with shore hardness 70 backed with gelatine 20 % (by mass) as a good candidate — it exhibited the most extensive similarities to FMJ impact defects detectable with the unaided eye in forensic morphological practice. The remaining materials assessed in this work are not recommended for use as ballistic skin simulants, due to either a lack of comparability to cases from forensic practice, or their partial – or total – failure in matching requirements for a simulant. The findings reported herein will be predominantly helpful in investigations aimed at identifying an appropriate skin simulant for wound ballistic investigation, particularly with regards to macromorphological comparability.

### Supplementary information

Below is the link to the electronic supplementary material.

Supplementary file1 (PPTX 13577 KB)

## Data Availability

The data that support the findings of this study are securely archived on a server of the Institute of Legal Medicine, University of Cologne, Germany, and can be made available upon request.
