# Supplementary material for: Skin simulants for wound ballistic investigation – an experimental study
Source: Int J Legal Med. 2024 Apr 3;138(4):1357–68. doi: 10.1007/s00414-024-03223-1 (PMC11164785; doi:10.1007/s00414-024-03223-1)
Supplement: Supplementary file 1 — Supplementary file1 (PPTX 13577 KB) [file 414_2024_3223_MOESM1_ESM.pptx]

## Slide 1
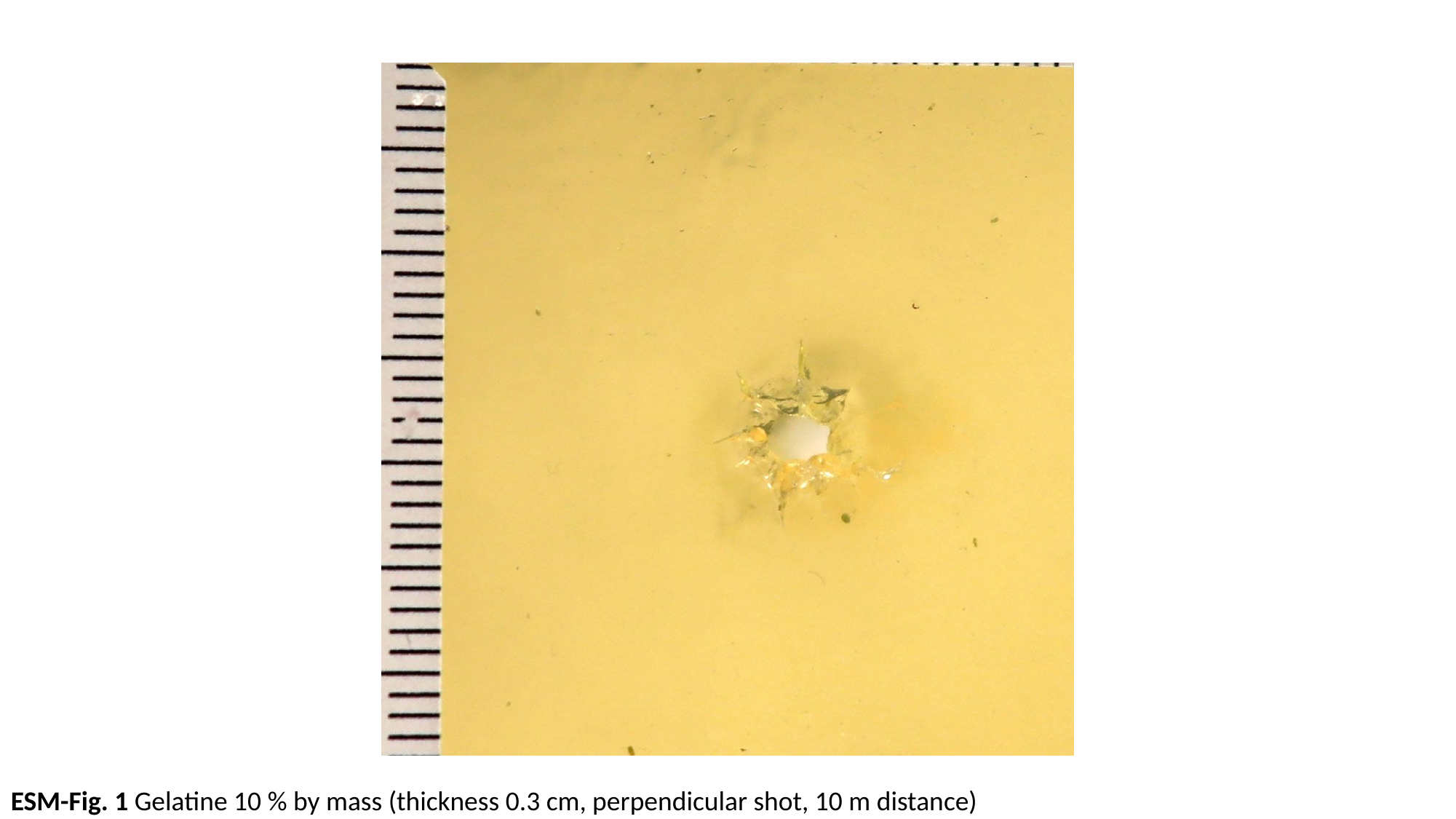

ESM-Fig. 1 Gelatine 10 % by mass (thickness 0.3 cm, perpendicular shot, 10 m distance)

## Slide 2
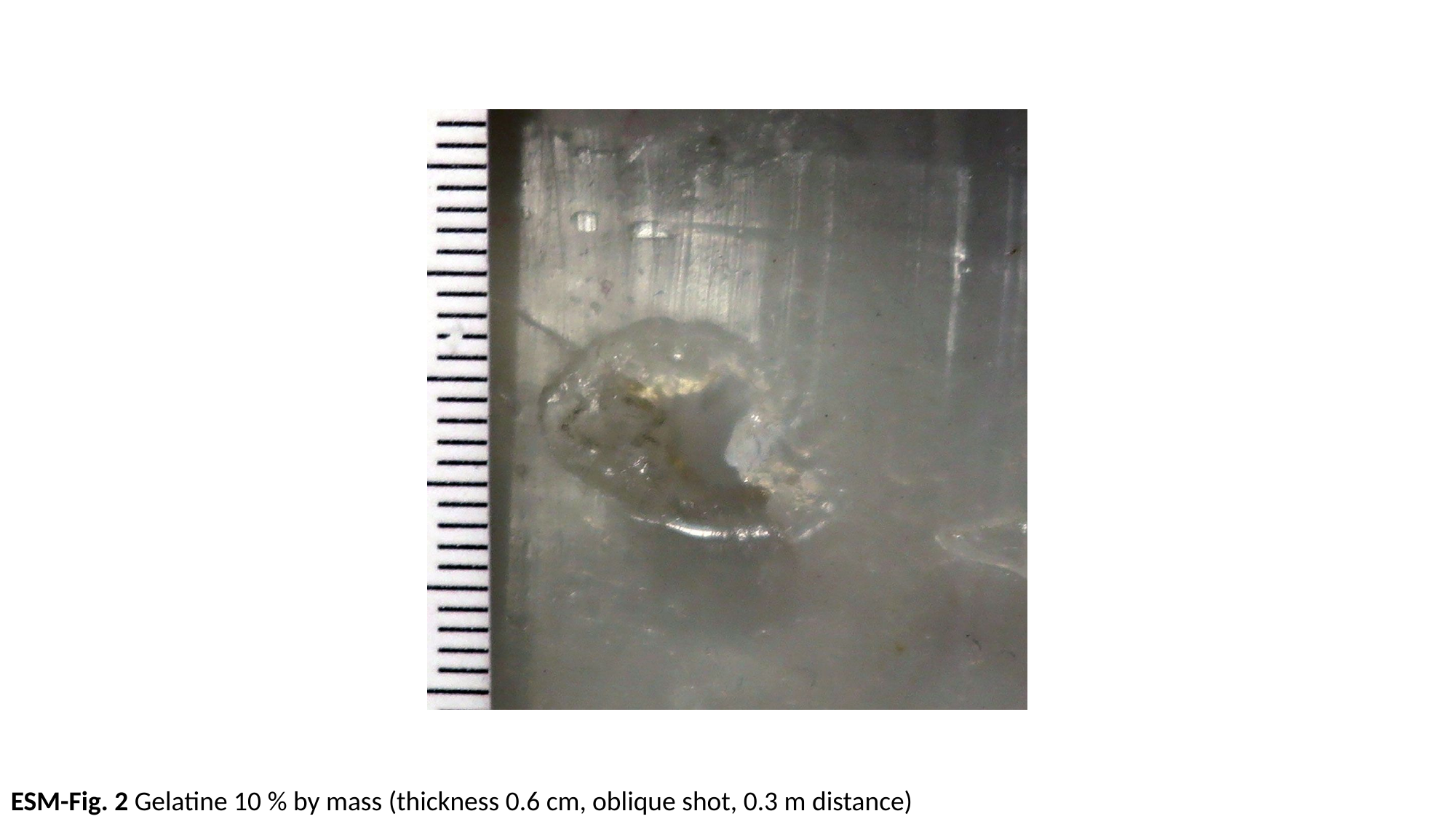

ESM-Fig. 2 Gelatine 10 % by mass (thickness 0.6 cm, oblique shot, 0.3 m distance)

## Slide 3
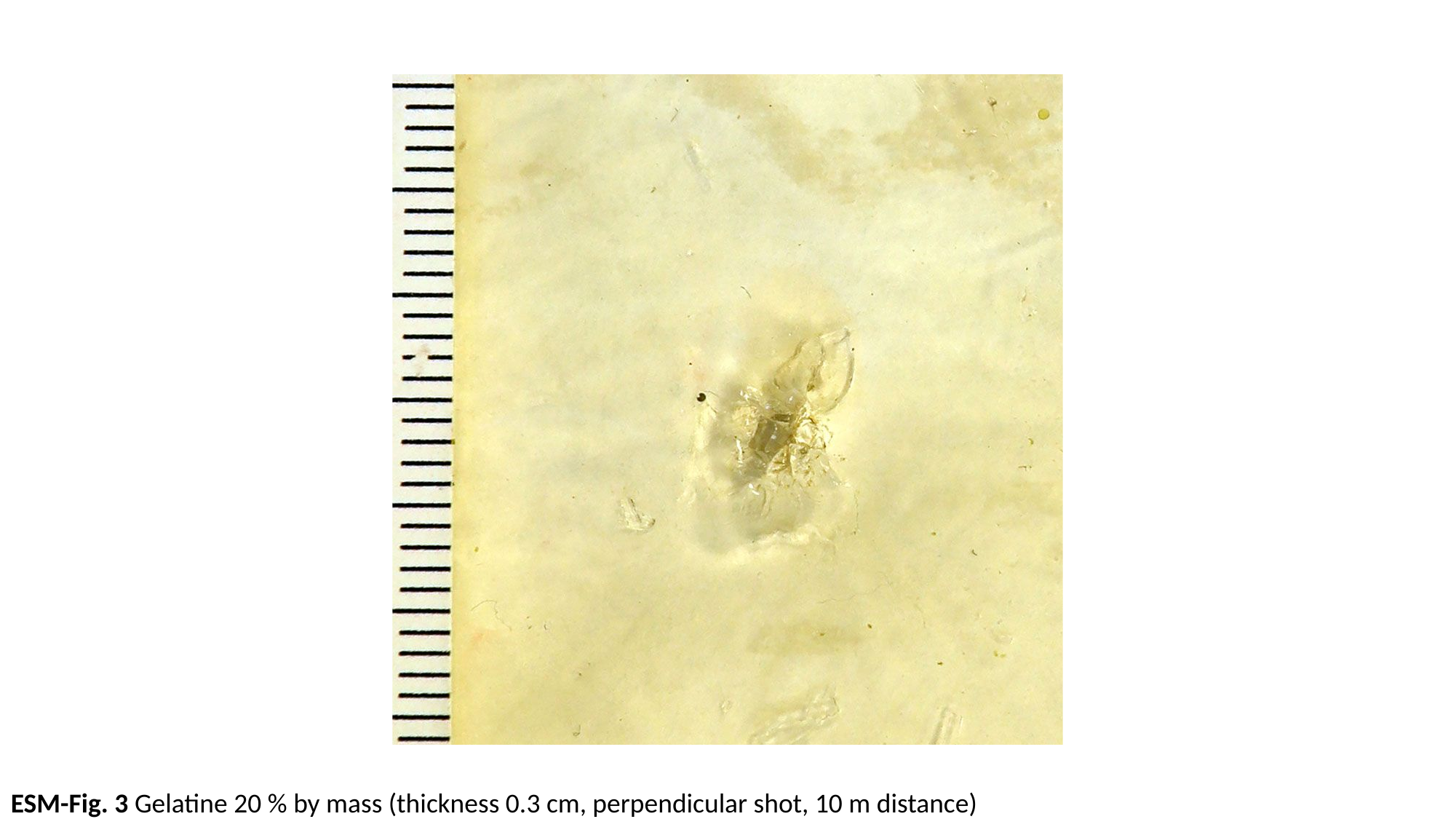

ESM-Fig. 3 Gelatine 20 % by mass (thickness 0.3 cm, perpendicular shot, 10 m distance)

## Slide 4
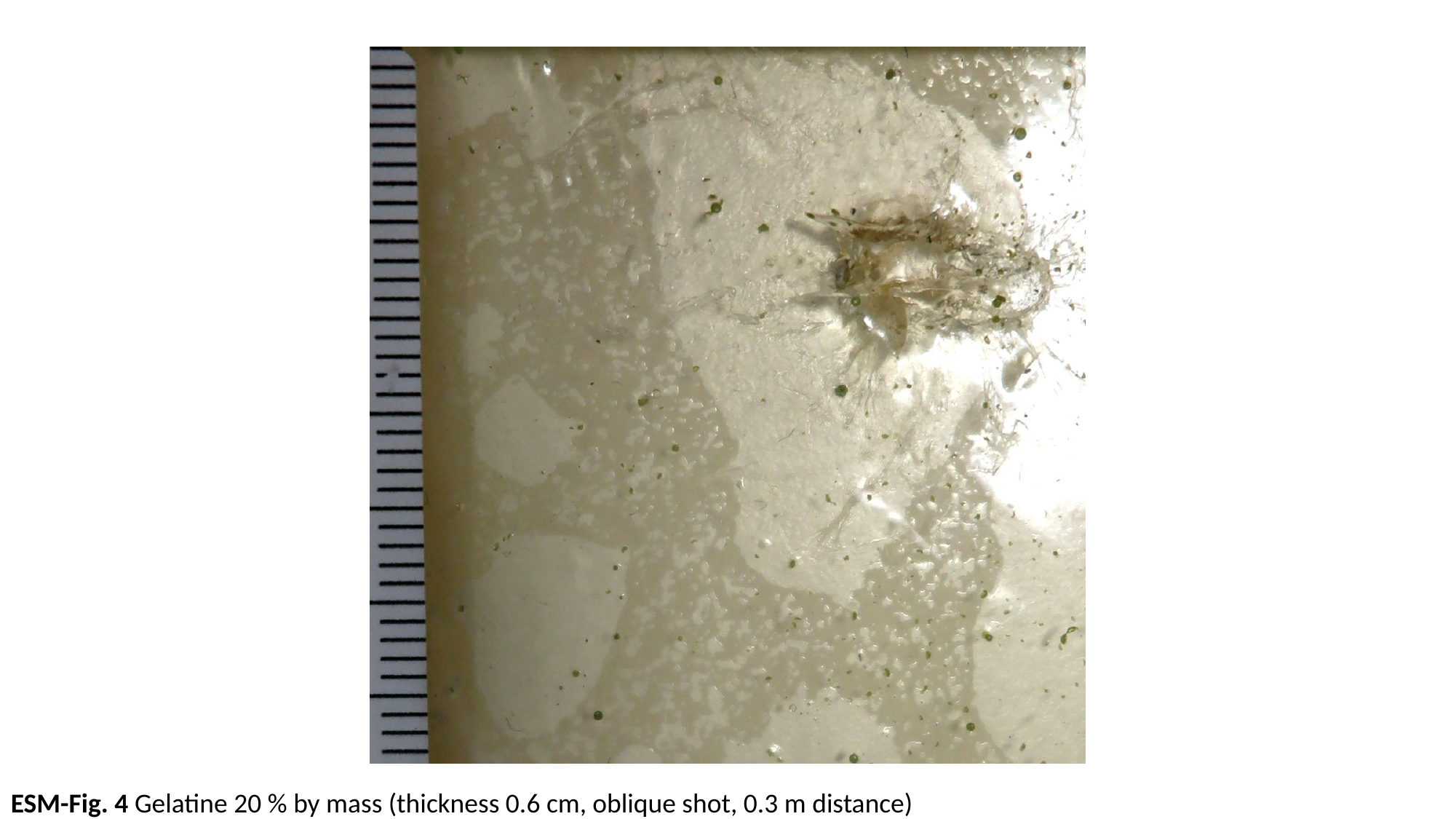

ESM-Fig. 4 Gelatine 20 % by mass (thickness 0.6 cm, oblique shot, 0.3 m distance)

## Slide 5
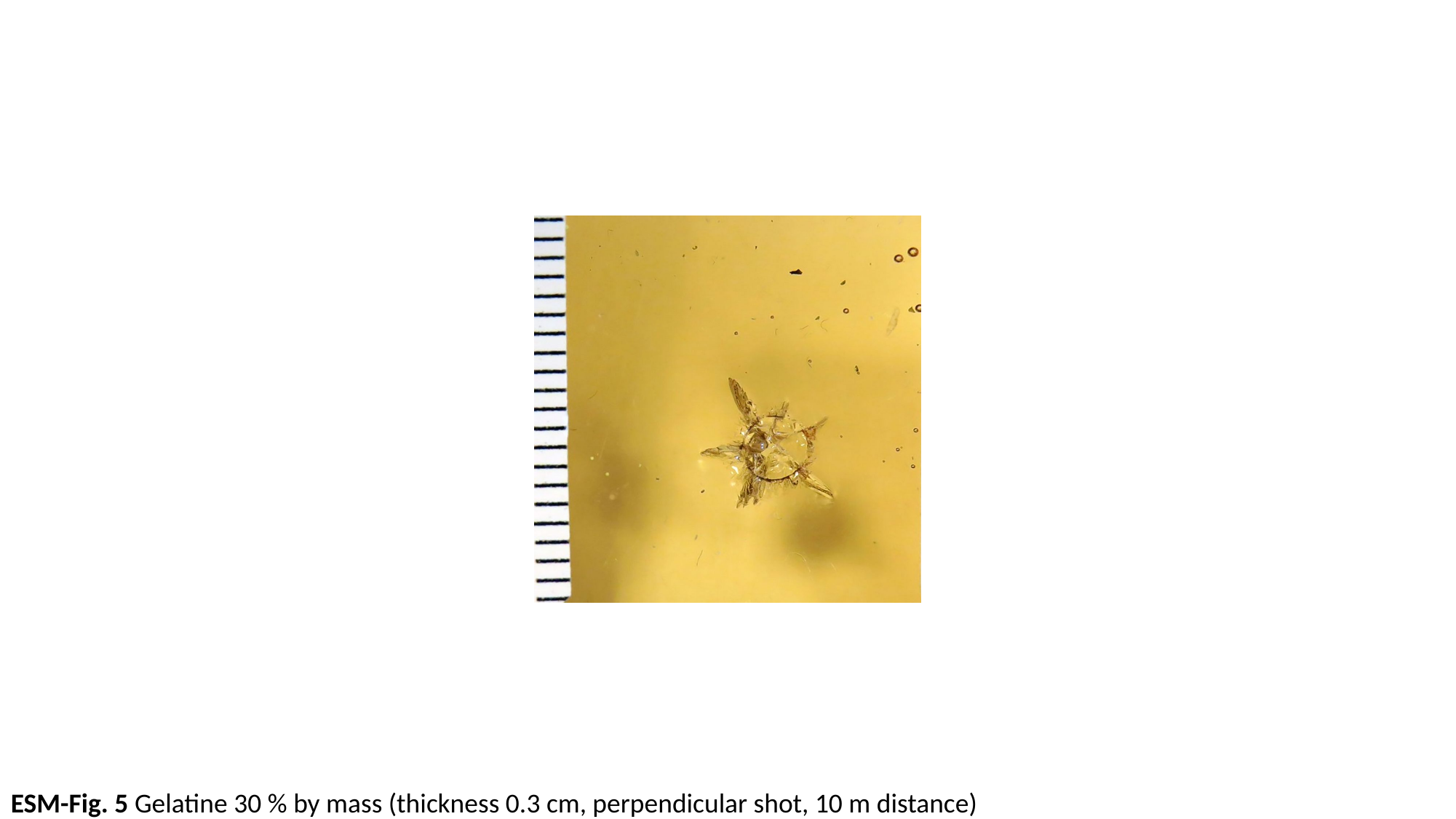

ESM-Fig. 5 Gelatine 30 % by mass (thickness 0.3 cm, perpendicular shot, 10 m distance)

## Slide 6
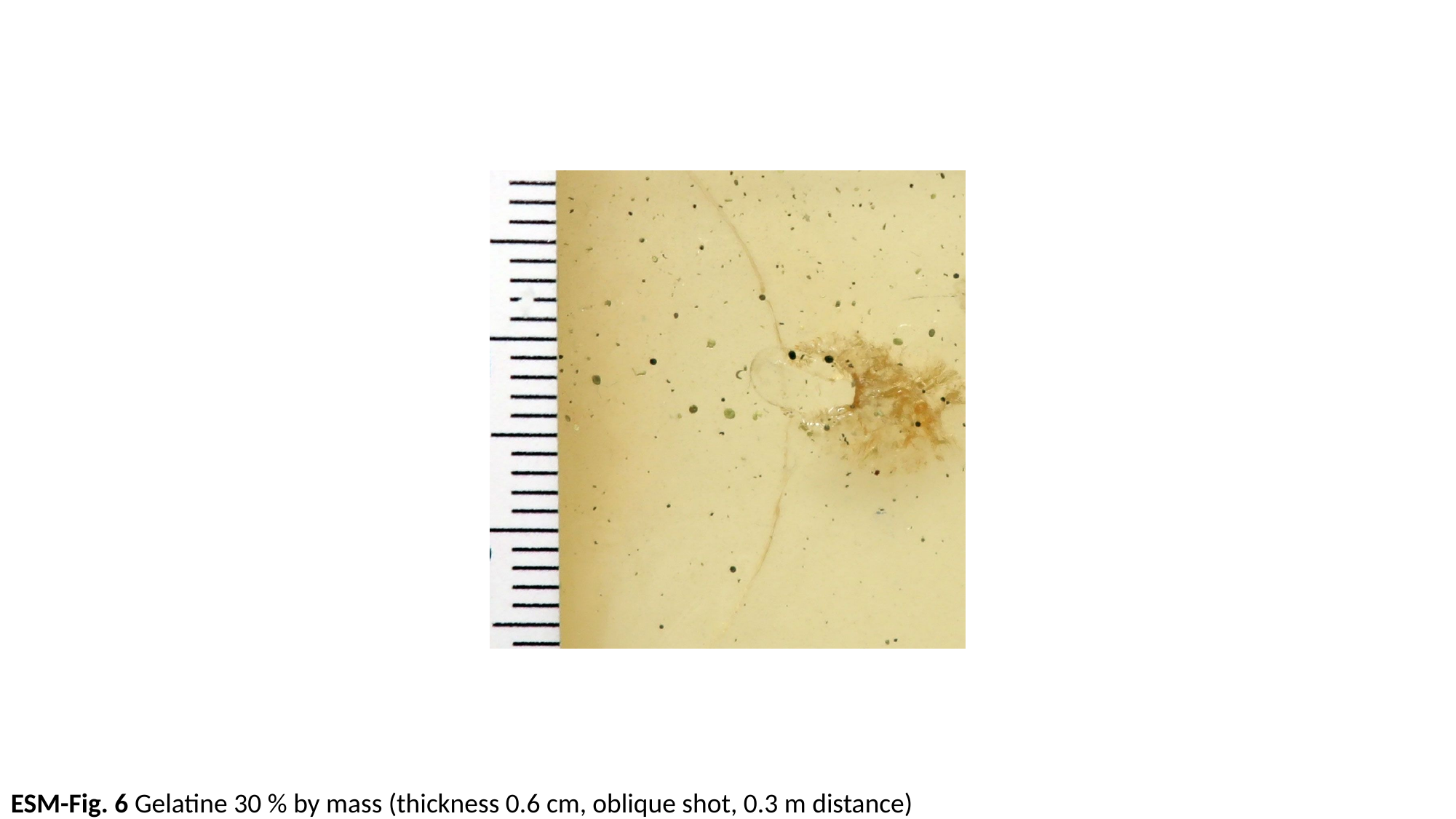

ESM-Fig. 6 Gelatine 30 % by mass (thickness 0.6 cm, oblique shot, 0.3 m distance)

## Slide 7
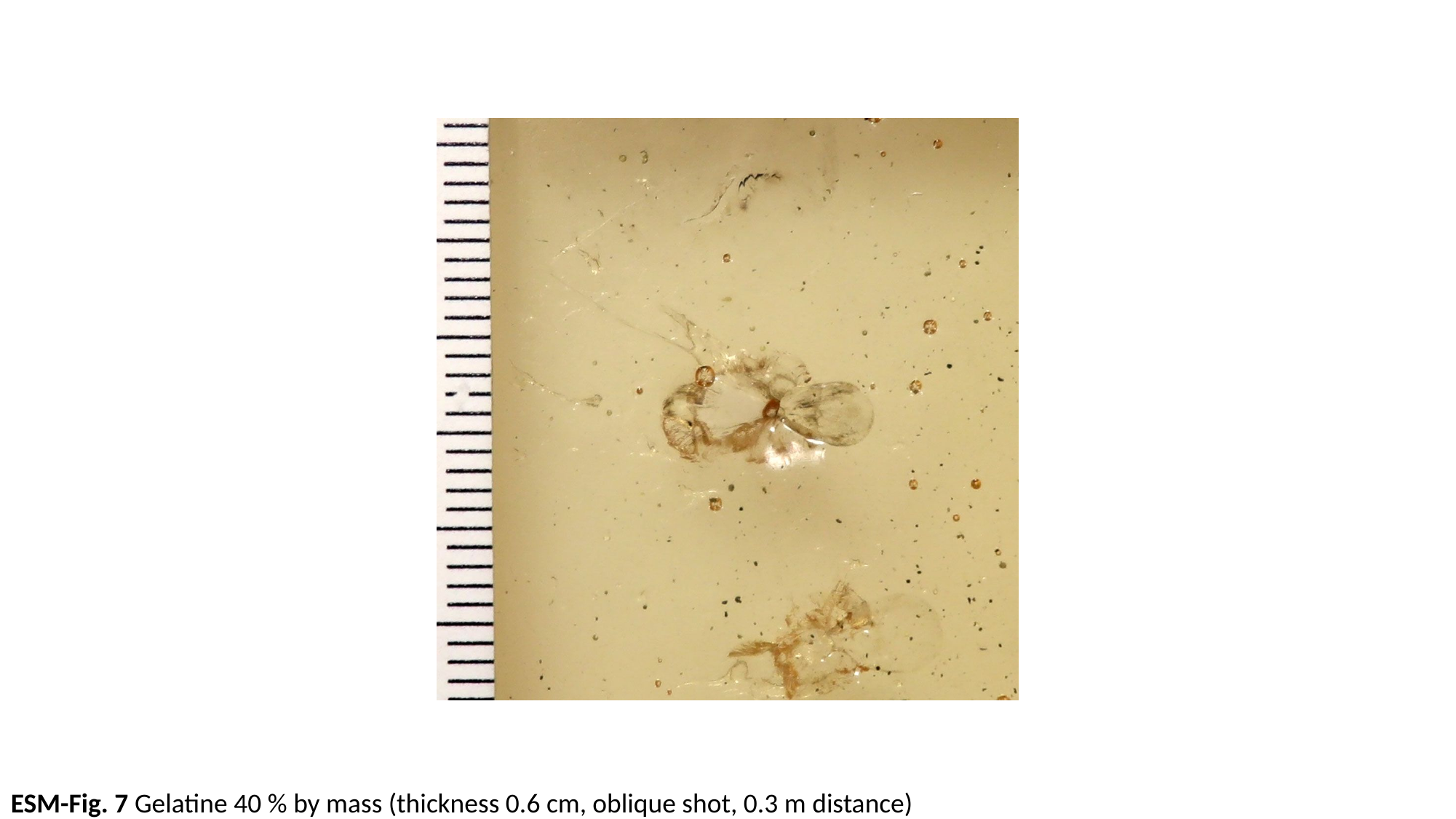

ESM-Fig. 7 Gelatine 40 % by mass (thickness 0.6 cm, oblique shot, 0.3 m distance)

## Slide 8
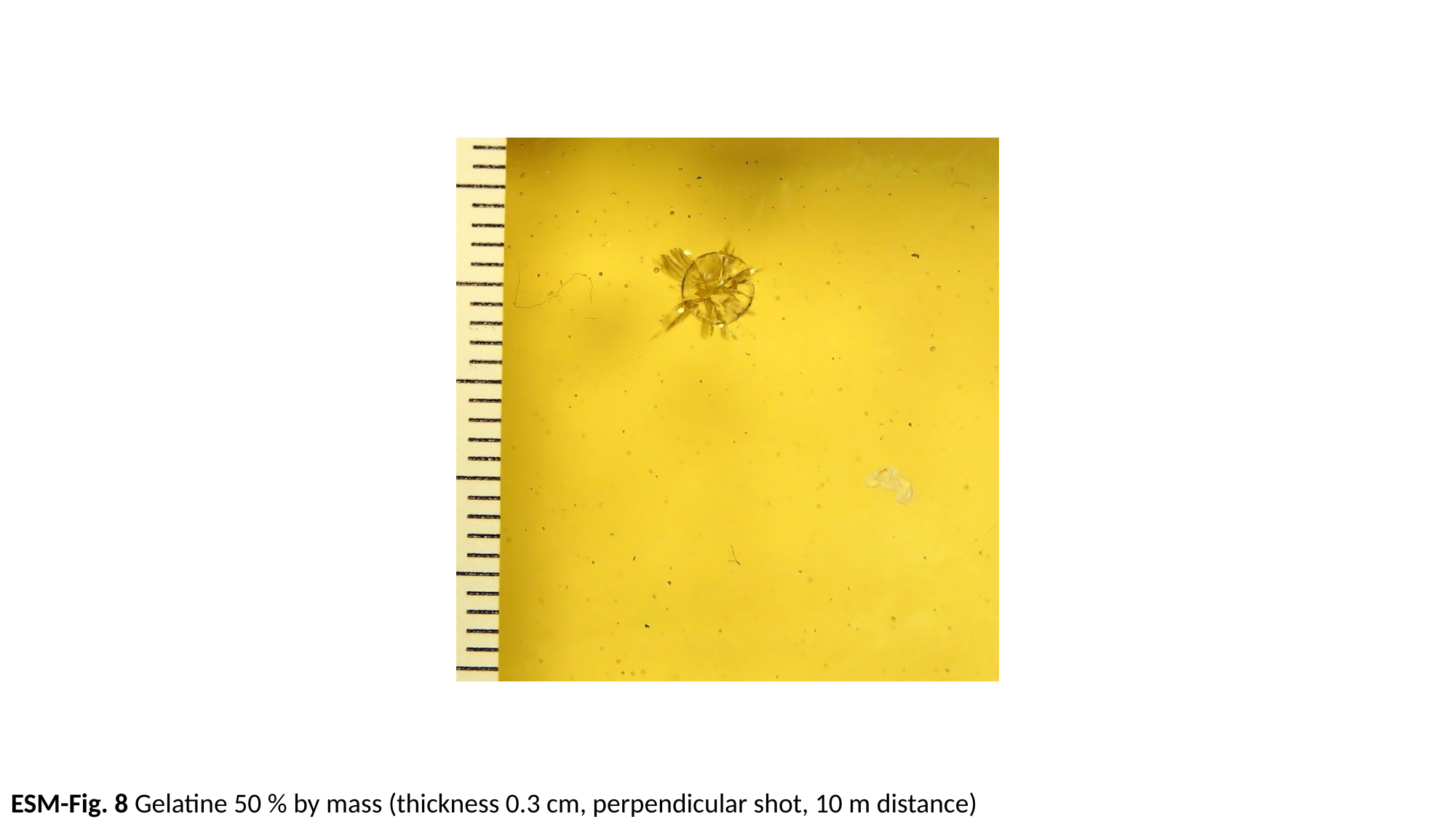

ESM-Fig. 8 Gelatine 50 % by mass (thickness 0.3 cm, perpendicular shot, 10 m distance)

## Slide 9
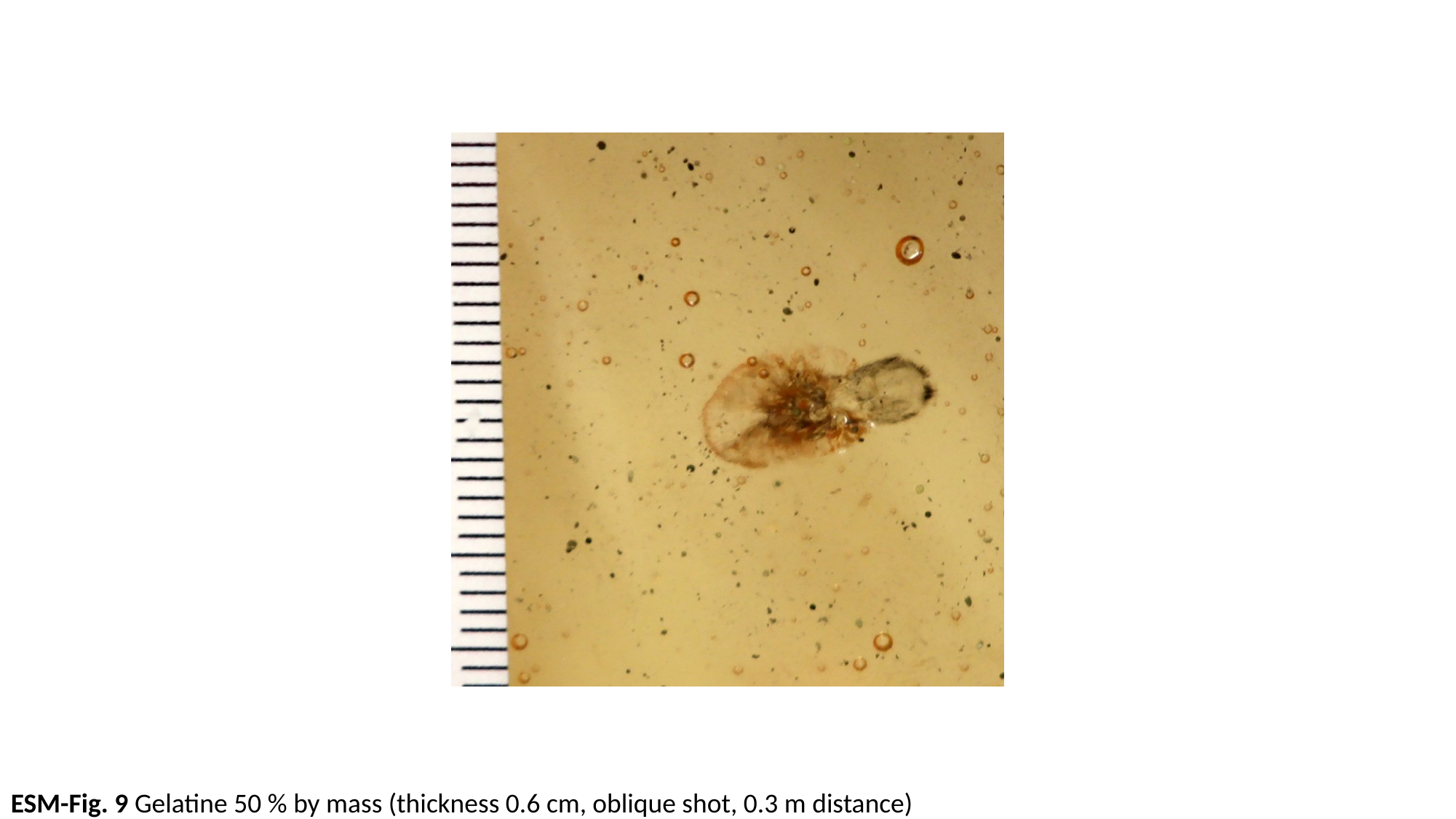

ESM-Fig. 9 Gelatine 50 % by mass (thickness 0.6 cm, oblique shot, 0.3 m distance)

## Slide 10
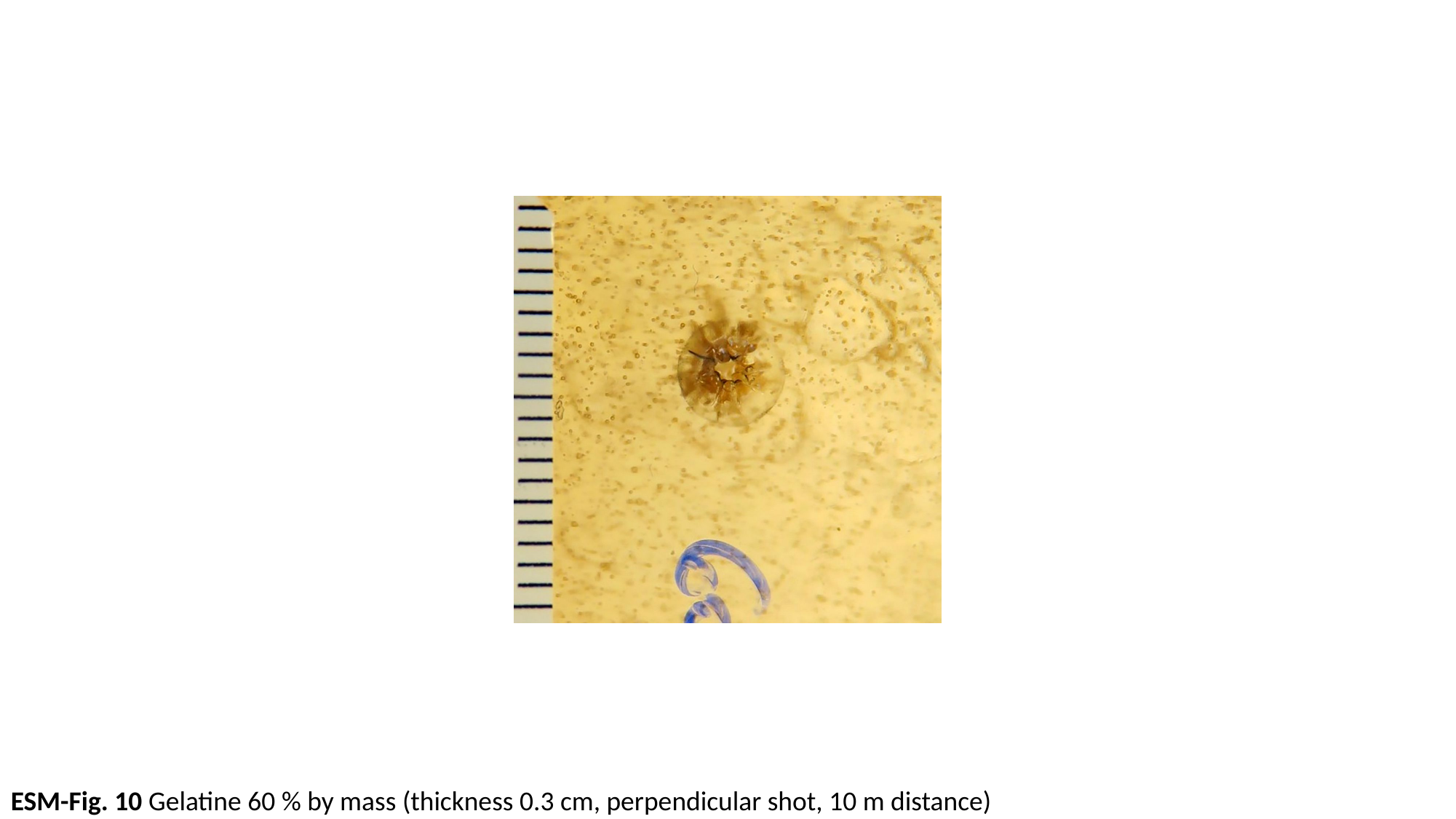

ESM-Fig. 10 Gelatine 60 % by mass (thickness 0.3 cm, perpendicular shot, 10 m distance)

## Slide 11
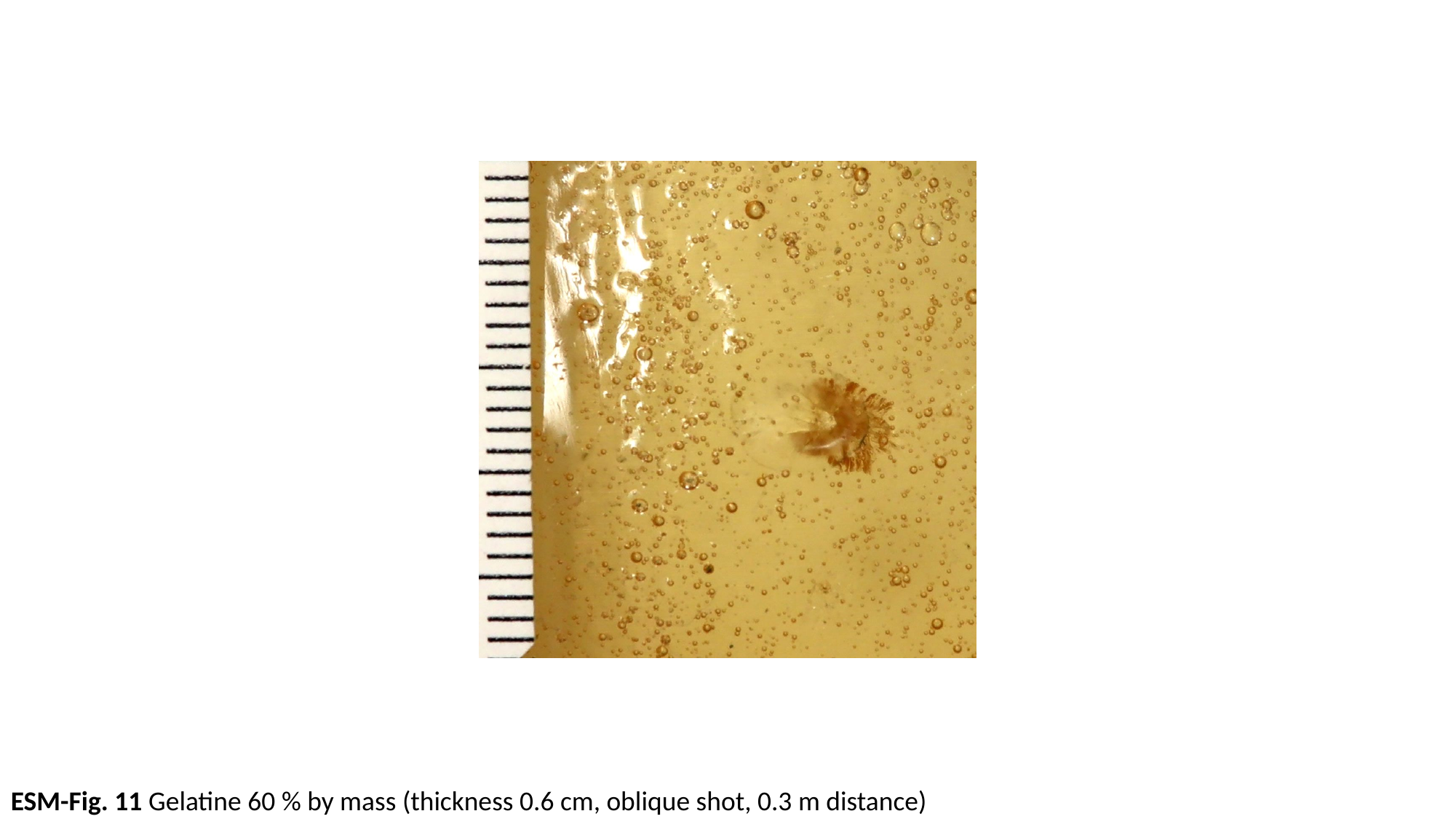

ESM-Fig. 11 Gelatine 60 % by mass (thickness 0.6 cm, oblique shot, 0.3 m distance)

## Slide 12
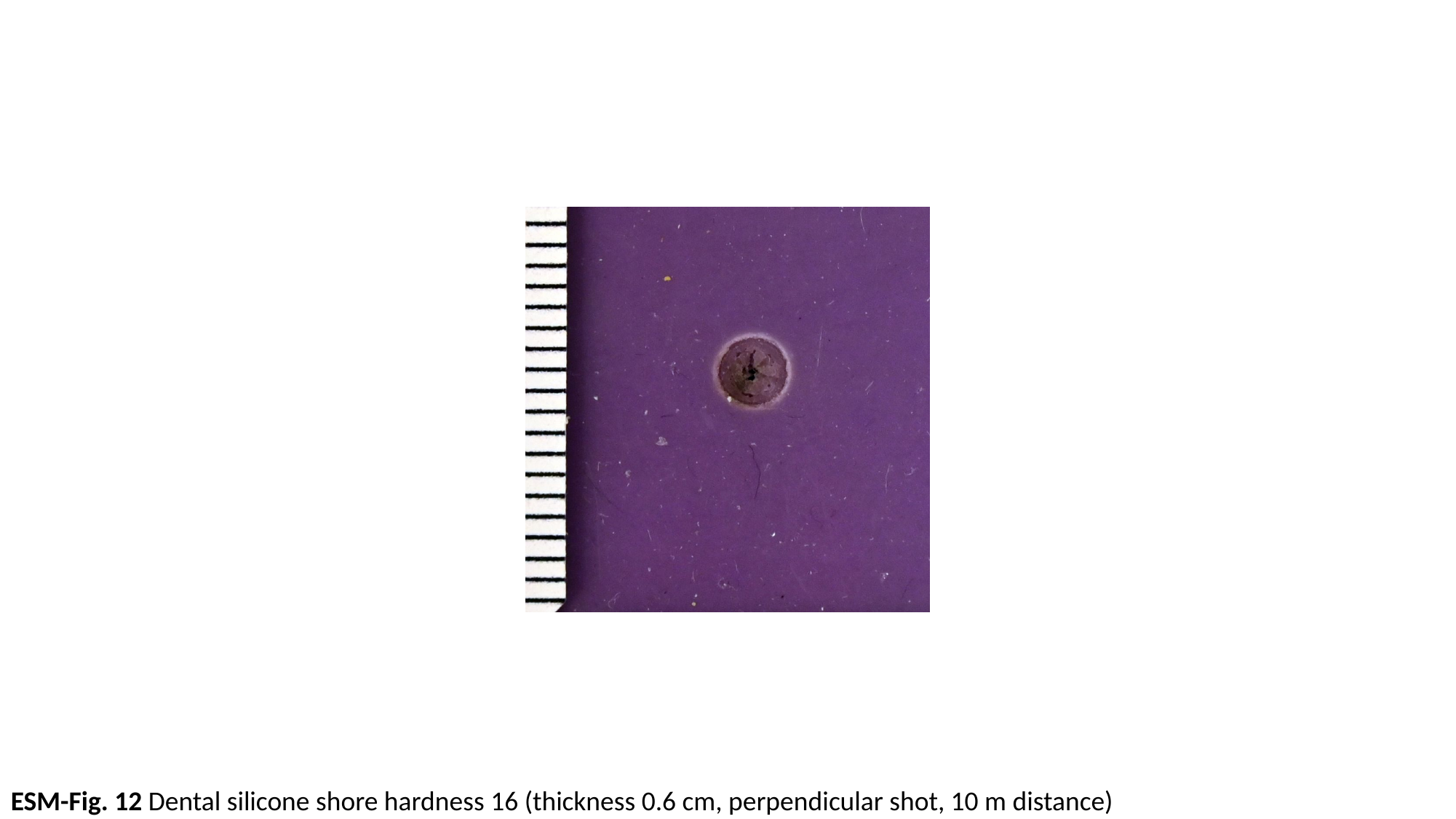

ESM-Fig. 12 Dental silicone shore hardness 16 (thickness 0.6 cm, perpendicular shot, 10 m distance)

## Slide 13
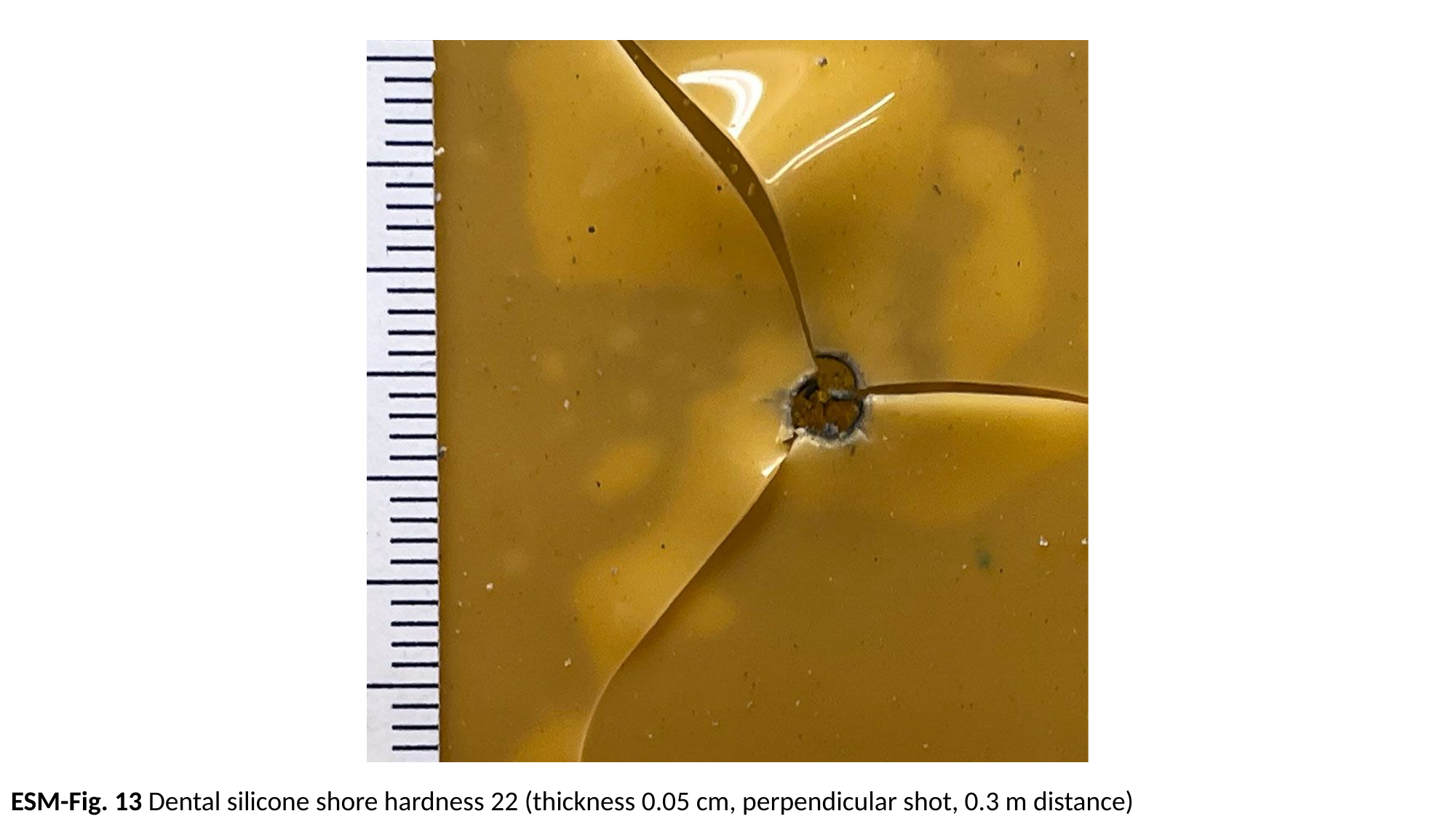

ESM-Fig. 13 Dental silicone shore hardness 22 (thickness 0.05 cm, perpendicular shot, 0.3 m distance)

## Slide 14
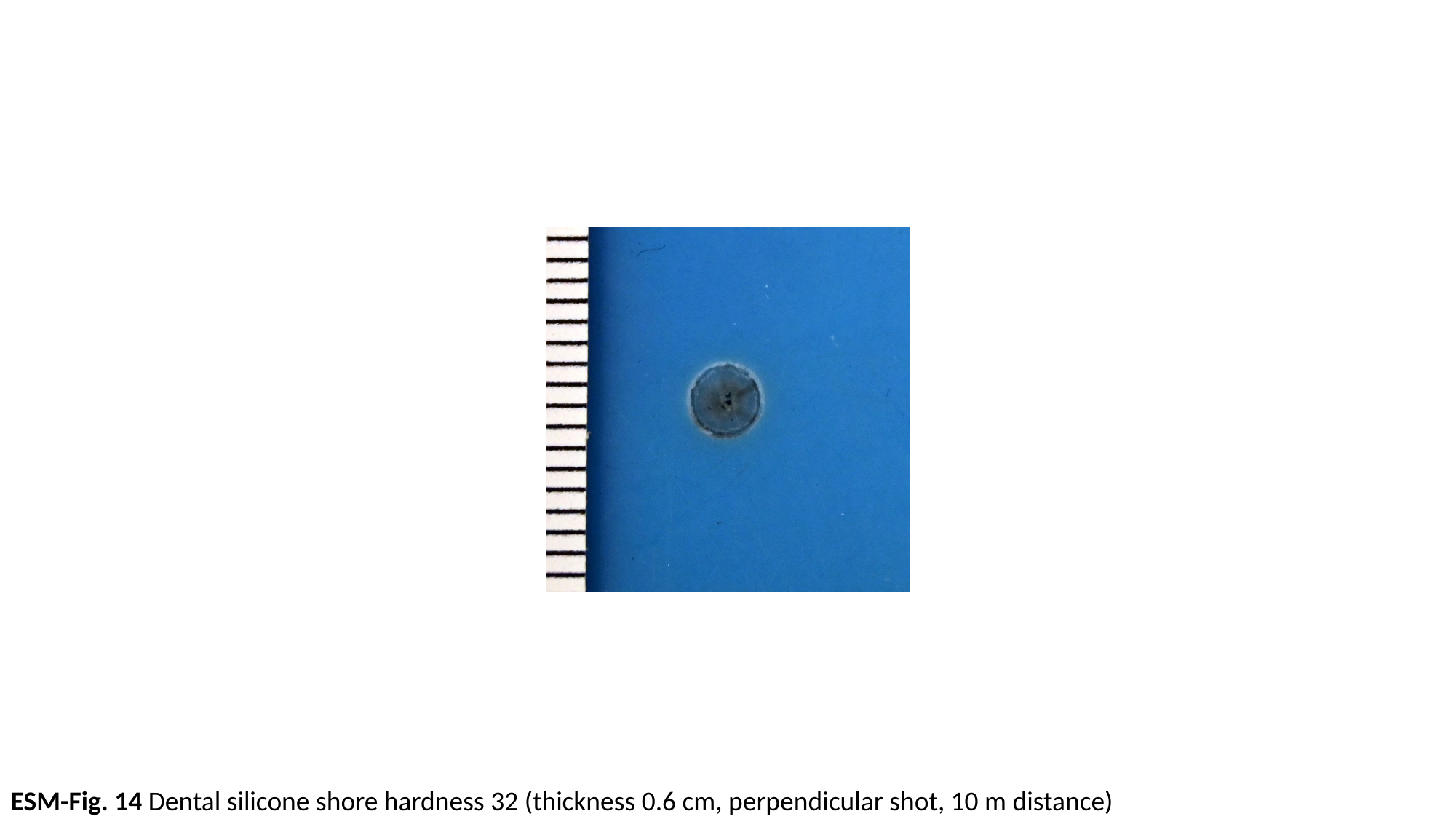

ESM-Fig. 14 Dental silicone shore hardness 32 (thickness 0.6 cm, perpendicular shot, 10 m distance)

## Slide 15
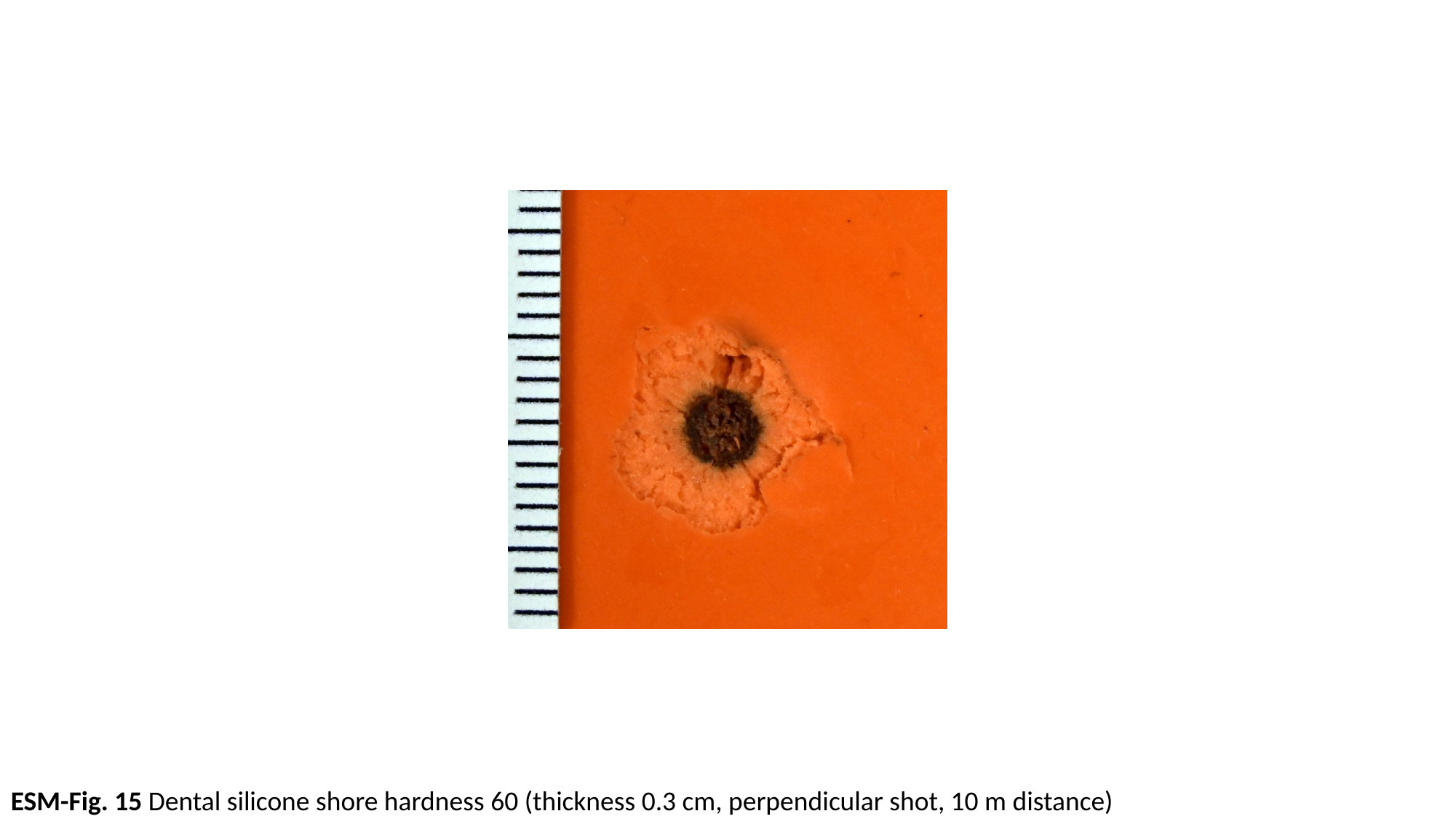

ESM-Fig. 15 Dental silicone shore hardness 60 (thickness 0.3 cm, perpendicular shot, 10 m distance)

## Slide 16
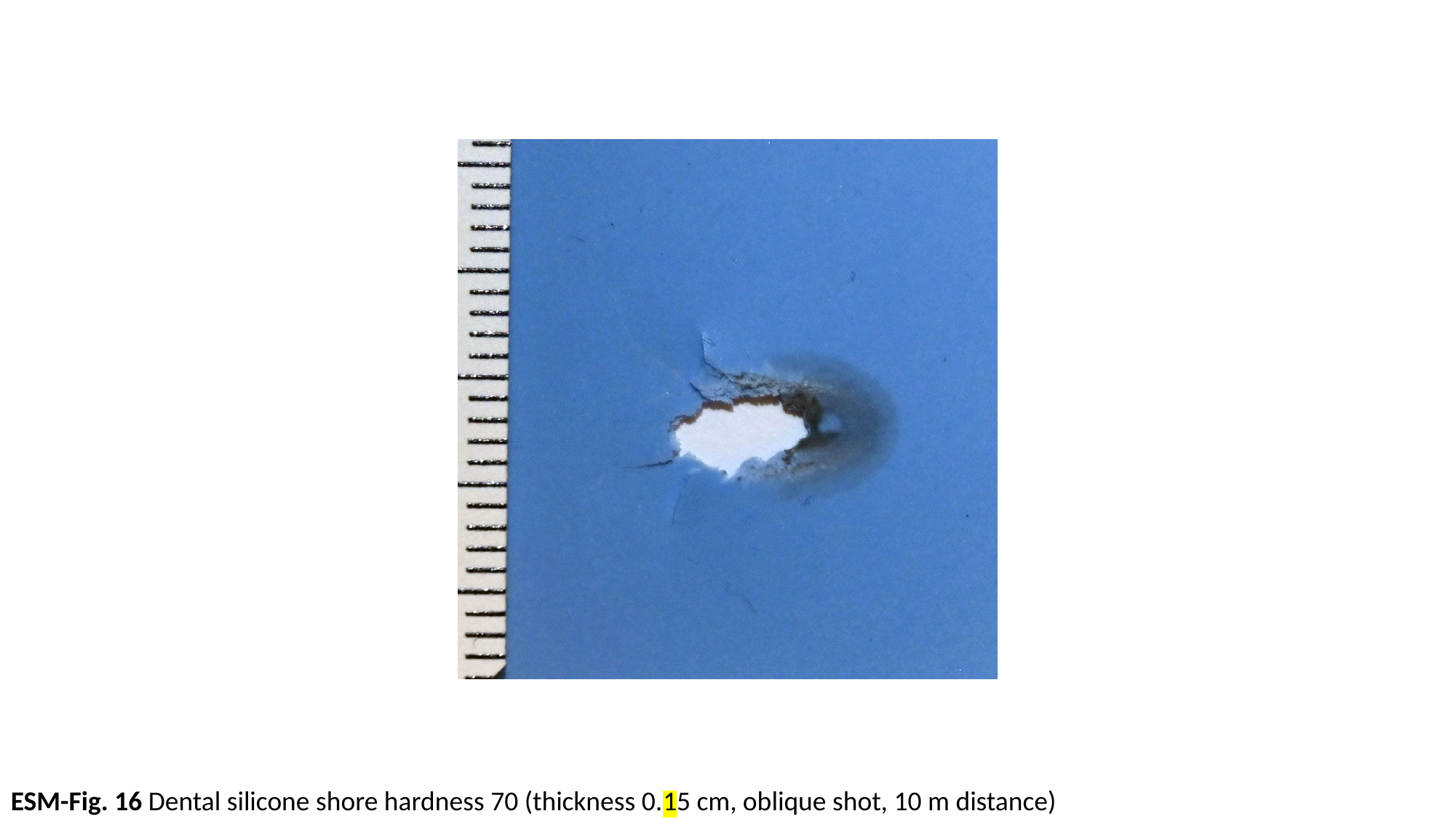

ESM-Fig. 16 Dental silicone shore hardness 70 (thickness 0.15 cm, oblique shot, 10 m distance)

## Slide 17
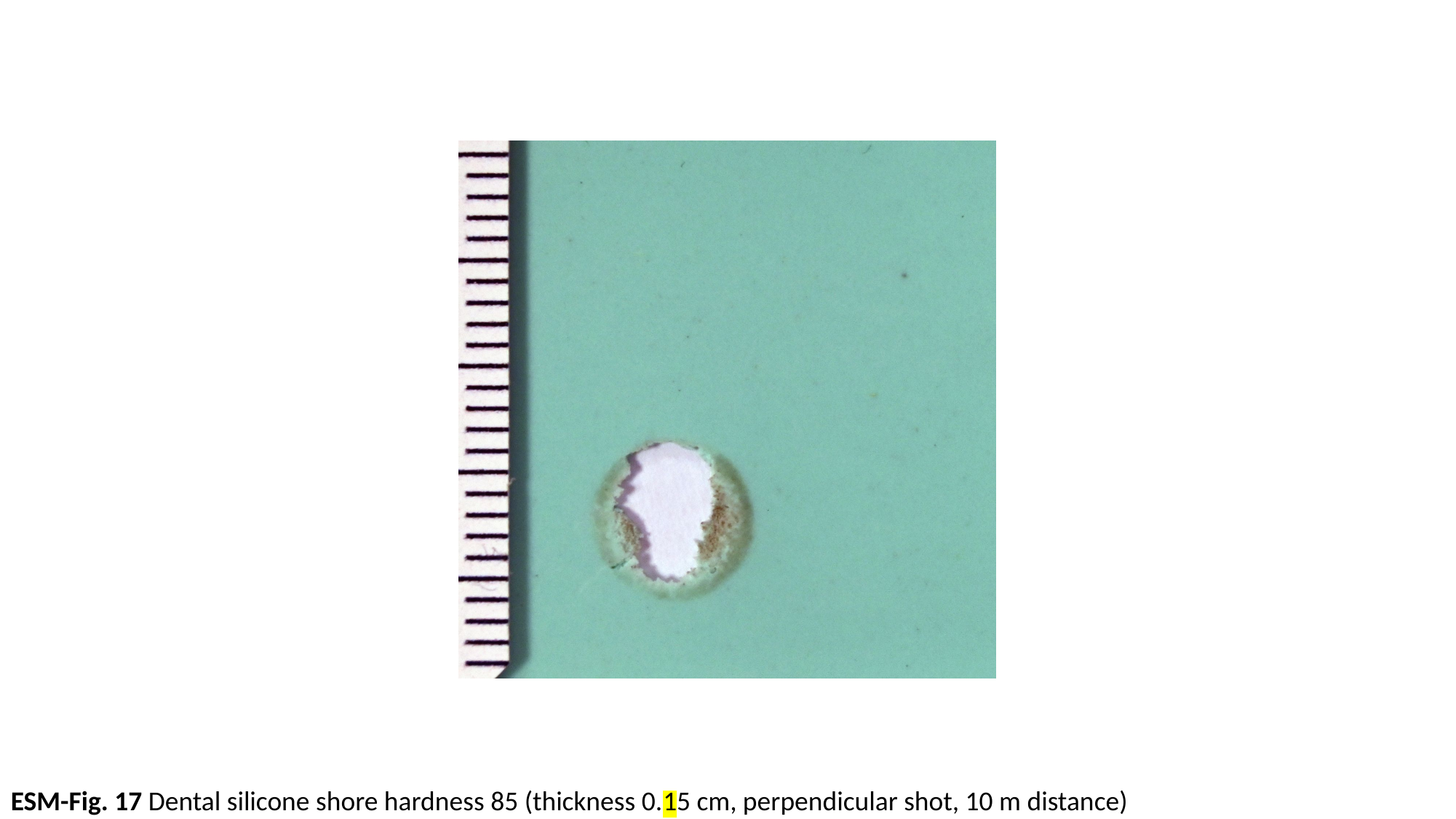

ESM-Fig. 17 Dental silicone shore hardness 85 (thickness 0.15 cm, perpendicular shot, 10 m distance)

## Slide 18
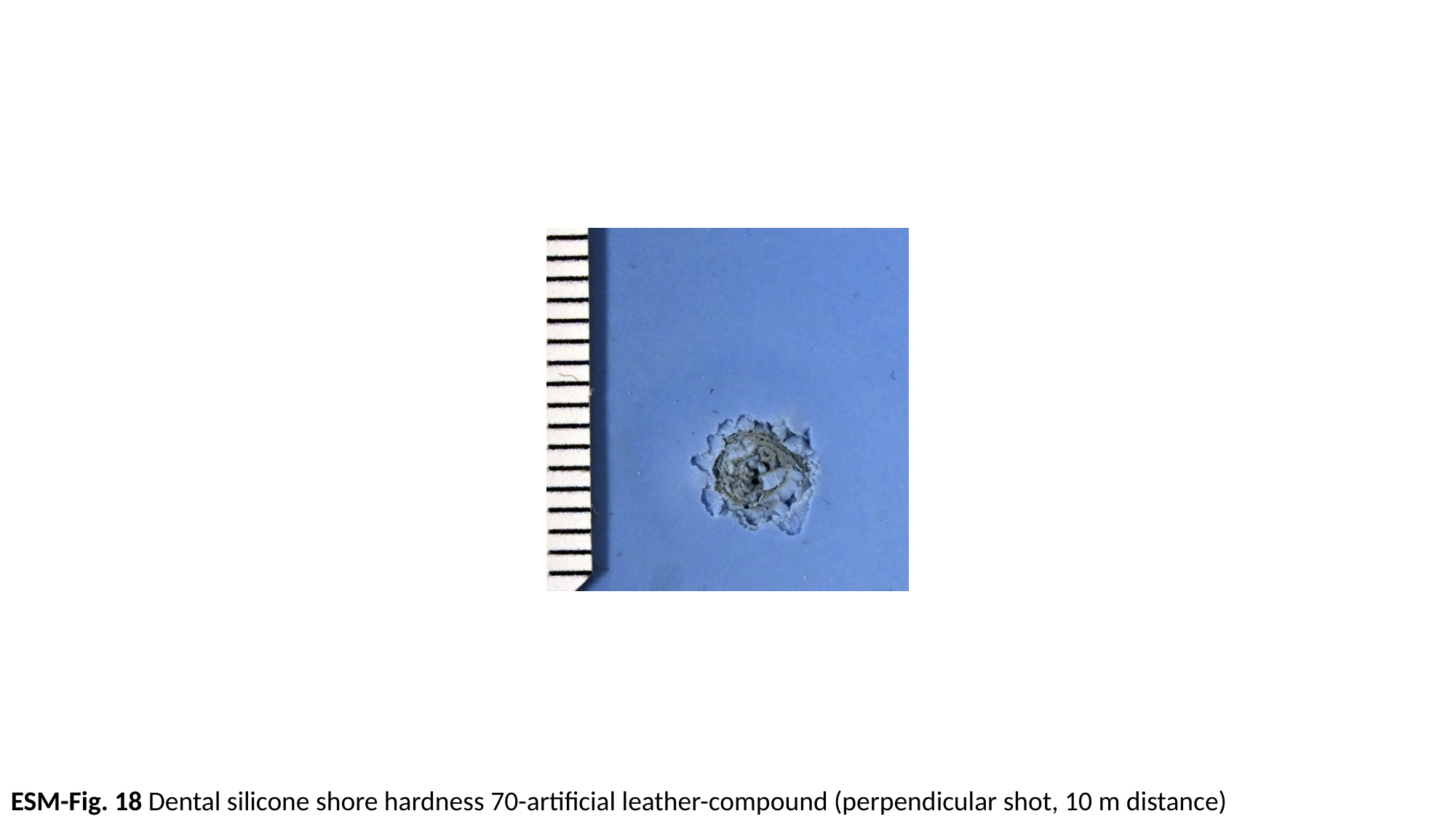

ESM-Fig. 18 Dental silicone shore hardness 70-artificial leather-compound (perpendicular shot, 10 m distance)

## Slide 19
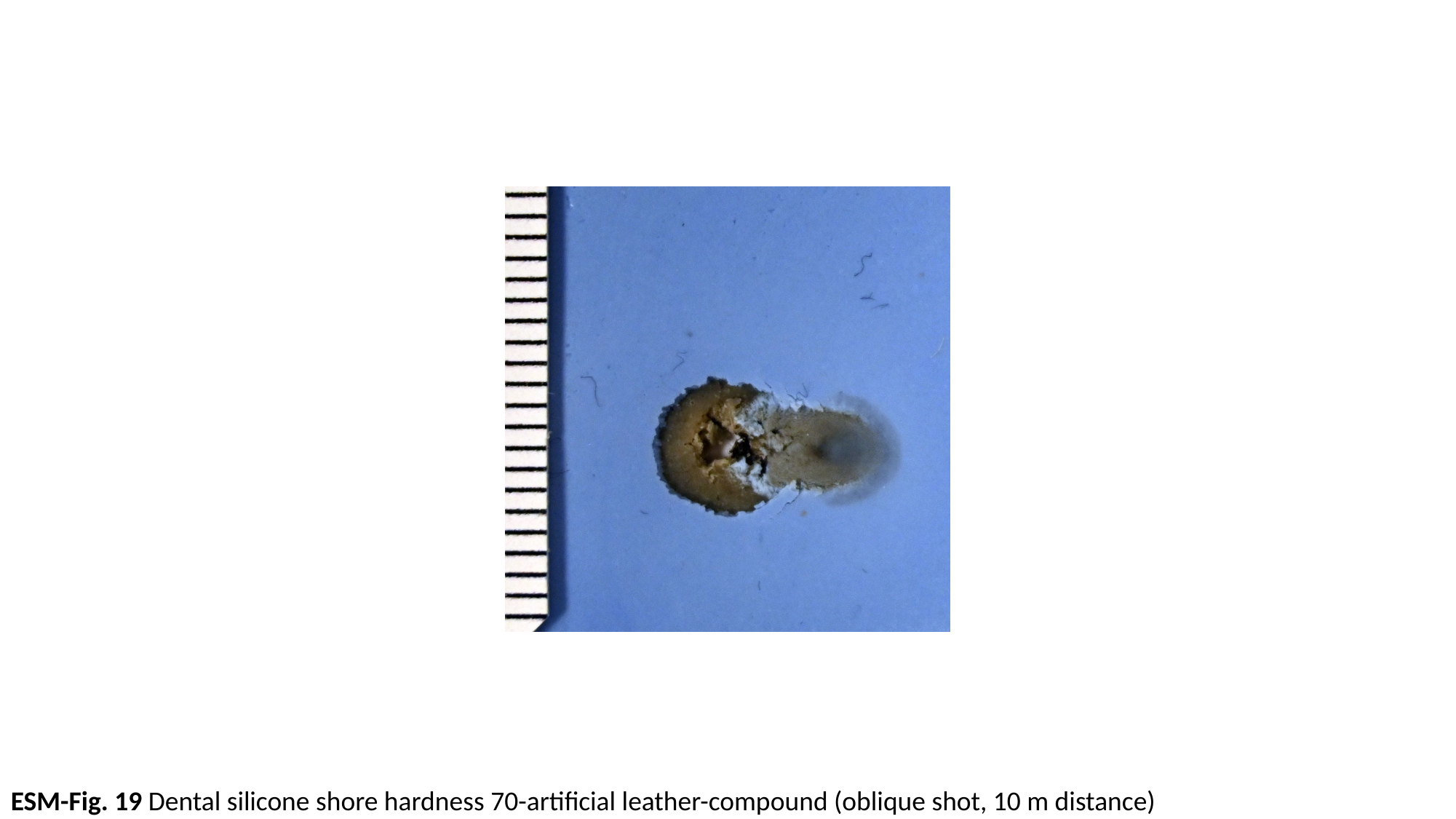

ESM-Fig. 19 Dental silicone shore hardness 70-artificial leather-compound (oblique shot, 10 m distance)

## Slide 20
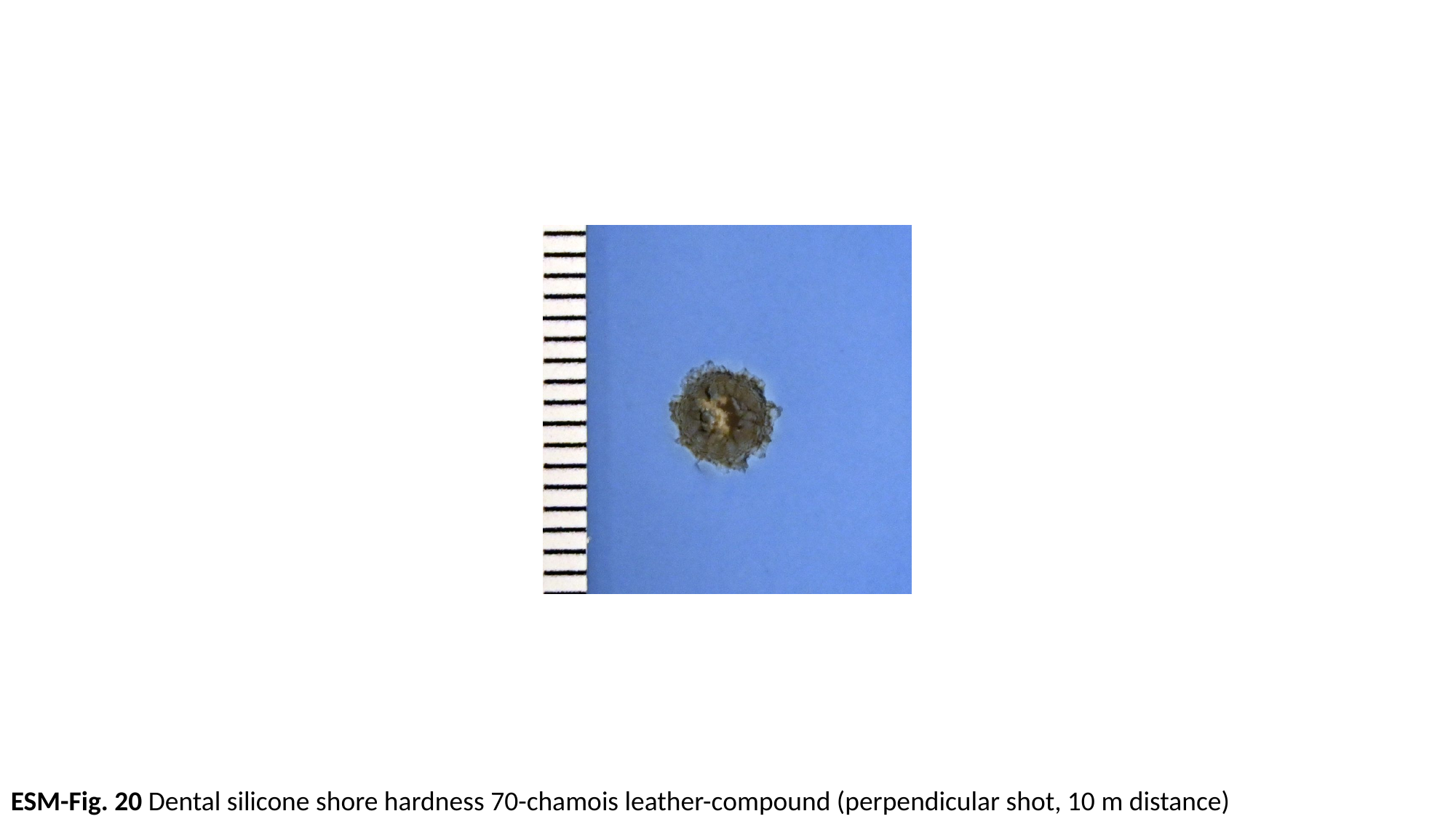

ESM-Fig. 20 Dental silicone shore hardness 70-chamois leather-compound (perpendicular shot, 10 m distance)

## Slide 21
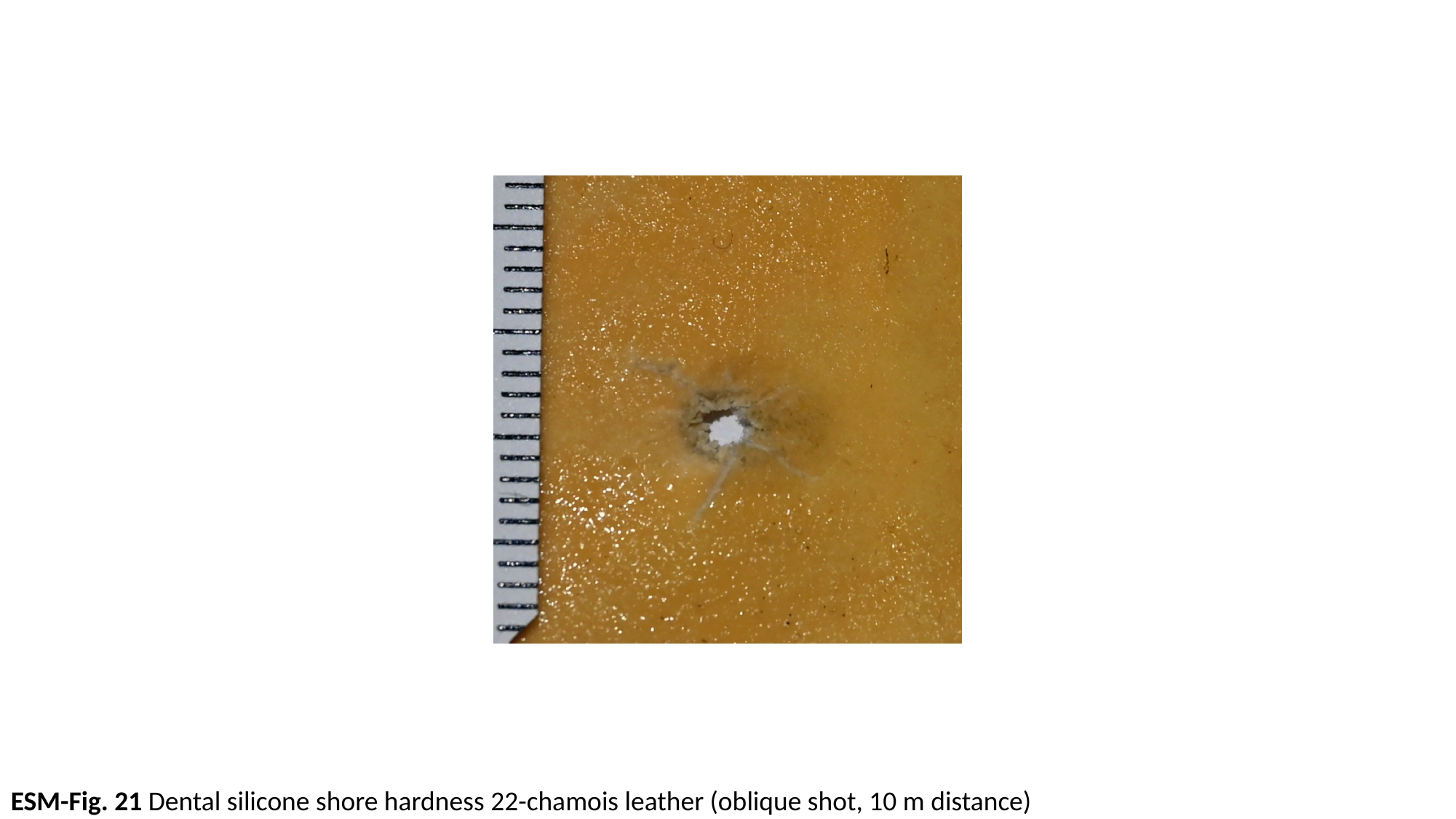

ESM-Fig. 21 Dental silicone shore hardness 22-chamois leather (oblique shot, 10 m distance)

## Slide 22
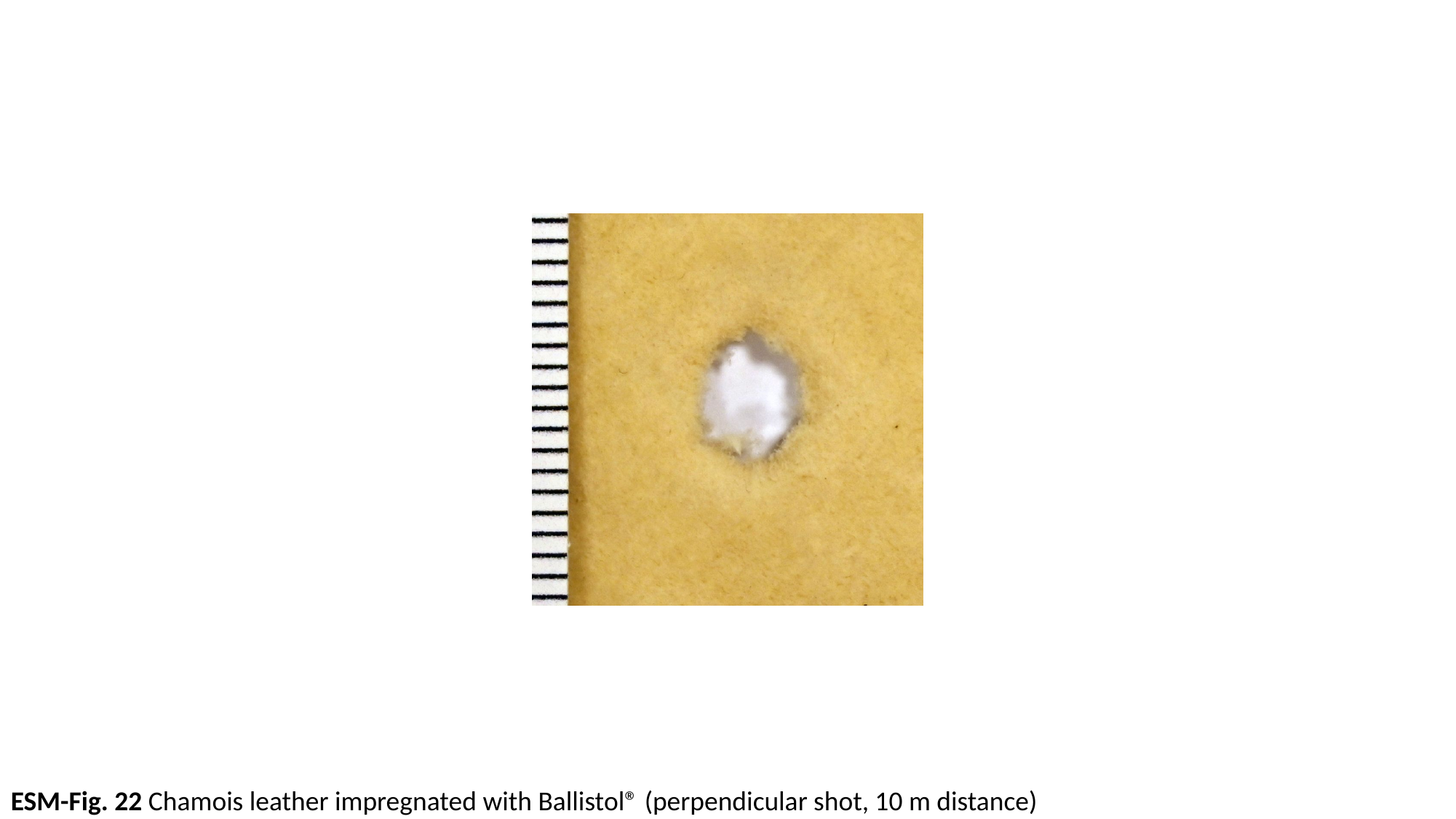

ESM-Fig. 22 Chamois leather impregnated with Ballistol® (perpendicular shot, 10 m distance)

## Slide 23
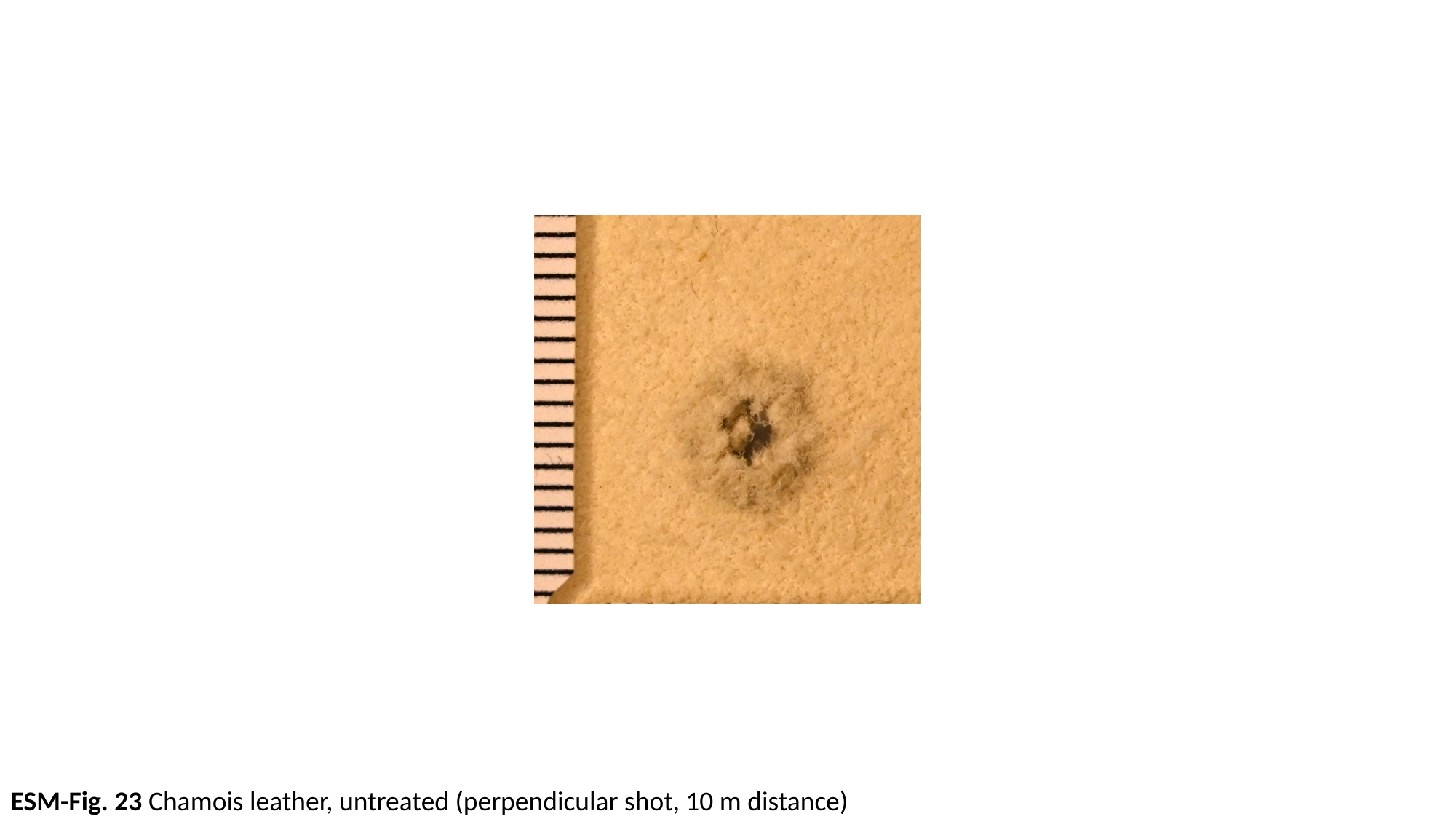

ESM-Fig. 23 Chamois leather, untreated (perpendicular shot, 10 m distance)

## Slide 24
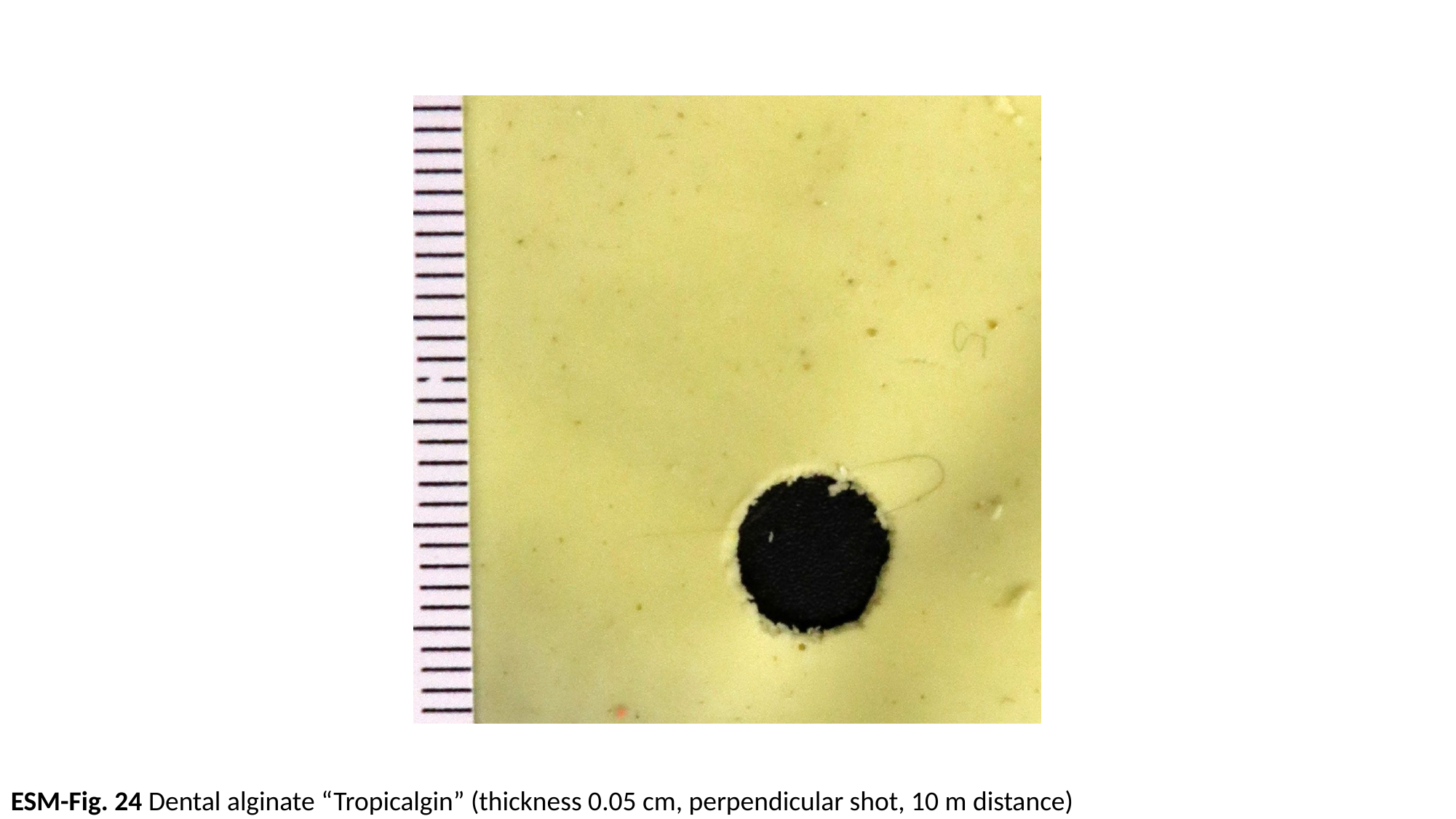

ESM-Fig. 24 Dental alginate “Tropicalgin” (thickness 0.05 cm, perpendicular shot, 10 m distance)

## Slide 25
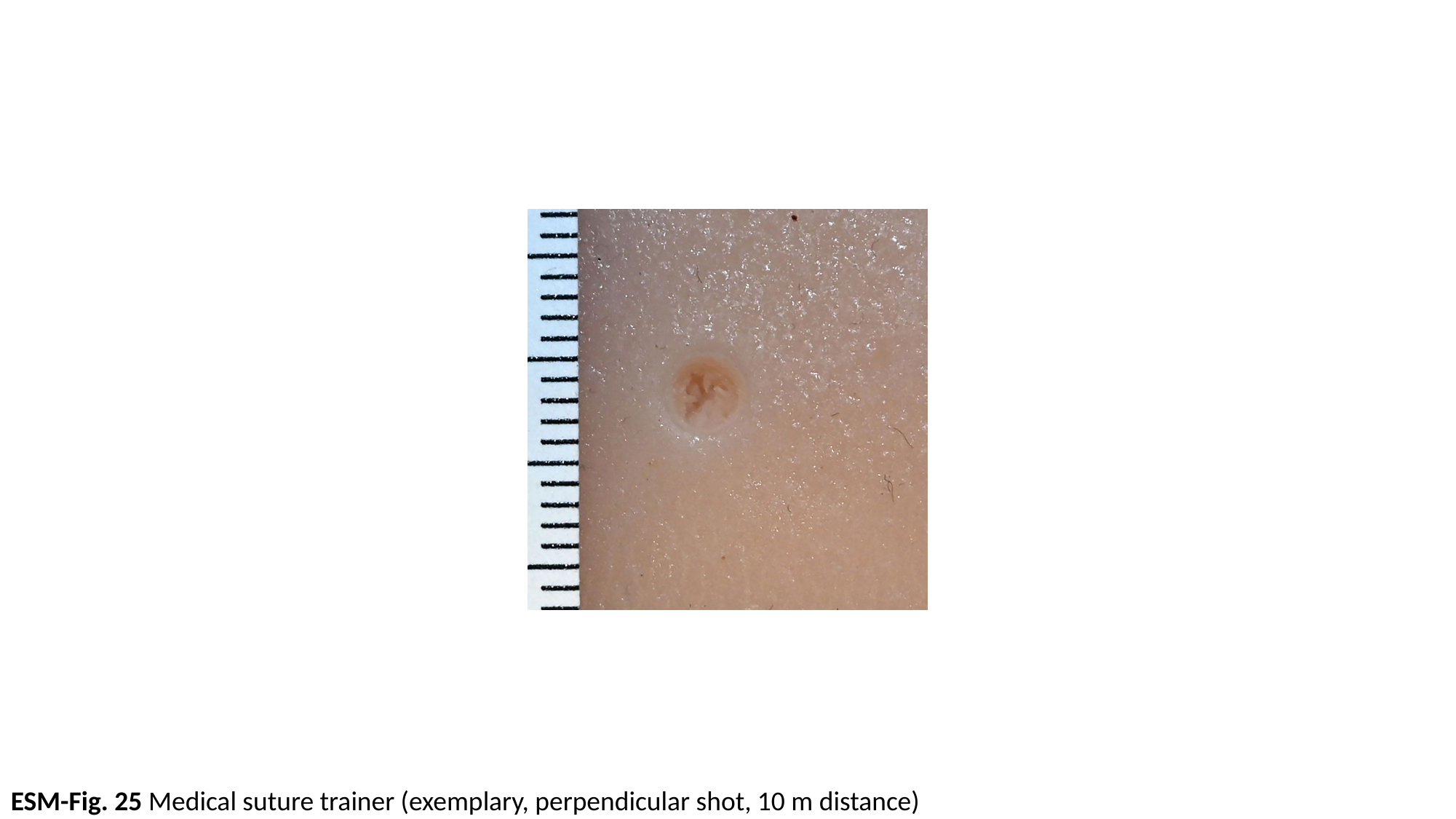

ESM-Fig. 25 Medical suture trainer (exemplary, perpendicular shot, 10 m distance)
